# Chronic Sarcoidosis: Diagnostic Difficulties and Search for New Criteria of Inflammatory Activity (A Case Report and Literature Review)

**DOI:** 10.3390/jcm13226974

**Published:** 2024-11-19

**Authors:** Anna Starshinova, Elizaveta Berg, Artem Rubinstein, Anastasia Kulpina, Igor Kudryavtsev, Dmitry Kudlay

**Affiliations:** 1 Department of Mathematics and Computer Science, St. Petersburg State University, St. Petersburg 199034, Russia; asya.starshinova@mail.ru; 2 Almazov National Medical Research Centre, St. Petersburg 197341, Russia; elevgberg@gmail.com (E.B.); igorek1981@yandex.ru (I.K.); 3 Department of Immunology, Institution of Experimental Medicine, St. Petersburg 197376, Russia; arrubin6@mail.ru; 4 Department of Pharmacology, Institute of Pharmacy, I.M. Sechenov First Moscow State Medical University, Moscow 119991, Russia; d624254@gmail.com; 5 Institute of Immunology, Moscow 115478, Russia; 6 Department of Pharmacognosy and Industrial Pharmacy, Faculty of Fundamental Medicine, Lomonosov Moscow State University, Moscow 119991, Russia

**Keywords:** chronic sarcoidosis, diagnosis of sarcoidosis, pulmonary sarcoidosis, cardiac sarcoidosis, skin sarcoidosis, neurosarcoidosis, ocular sarcoidosis, autoimmune inflammation, biomarkers

## Abstract

Sarcoidosis is a systemic inflammatory disease with an unknown etiology and a wide range of clinical manifestations. The incidence of sarcoidosis ranges from approximately 1 to 15 cases per 100,000 individuals per year worldwide. The significant variability in clinical presentations and target organs, as well as concomitant diseases, greatly complicates diagnosis. We analyzed articles in PubMed, Scopus, Cochrane Library, and Embase, where databases were searched using the keywords “chronic sarcoidosis”, “diagnosis of sarcoidosis”, “course of sarcoidosis”, “pulmonary sarcoidosis”, “cardiac sarcoidosis”, “skin sarcoidosis”, “neurosarcoidosis”, “ocular sarcoidosis”, and “autoimmune inflammation”. Studies on the course and diagnosis of sarcoidosis with a deep search of ten years were included. In this review, we present an analysis of publications on the course and diagnosis of chronic sarcoidosis, as well as a clinical case. We have noted that the diagnosis of chronic sarcoidosis is particularly difficult due to the lack of specific biomarkers or their combination. The development and introduction of new diagnostic criteria for this disease will contribute to increasing the level of efficiency, not only of the diagnostic complex, but also the prognosis of the development and course of the pathological process. Conclusion: For the most accurate diagnosis and determination of prognosis, the existence of a single immunological or imaging marker with sufficient sensitivity and specificity is necessary.

## 1. Introduction

To date, sarcoidosis remains a systemic disease, and its etiology is unknown. The disease is characterized by a wide range of clinical and radiological manifestations, with granulomatous lesions in various organs and systems, which lead to the development of severe complications. The main diagnostic criteria for sarcoidosis remain epithelioid-cellular non-caseating granulomas, detected during biopsy in various tissues and organs, mainly in the lungs and mediastinal lymph nodes. There are no clear statistics on the incidence of sarcoidosis worldwide. However, according to various authors, the incidence of sarcoidosis in different regions of the world varies from 1 to 15 per 100,000 individuals. The lowest rates are found in East Asia (0.5–1 per 100,000), average rates in North America and Australia (5–10 per 100,000), and the highest rates in Northern Europe (Scandinavia) (11–15 per 100,000) [1]. In the Russian Federation, the epidemiology of sarcoidosis also differs greatly depending on the region. According to an analysis of publications by A.A. Vizel, as of 2017, the highest prevalence is observed in the Republic of Karelia (73 per 100,000) and the lowest—in the Amur region (8.2 per 100,000) [1].

According to published data, in the United States, the highest prevalence of the disease is found among women of the Negro race (71 per 100,000), which may be due to racial genetic differences and the influence of socioeconomic environmental factors [2].

Sarcoidosis is more common in people younger than 40 years, with the peak incidence occurring between the ages of 25 and 40 years [3]. However, the disease can be diagnosed at any age.

One of the most reliable correlations between the influence of environmental factors and the development of sarcoidosis is associated with occupational exposure to insecticides, both in agricultural and industrial settings. However, agricultural workers are also exposed to other chemical aerosols, including grains, silicates, animal proteins, fungi, bacteria, mycotoxins, and endotoxins. An interesting fact was the published data on the registration of a large number of cases of sarcoidosis in people exposed to the attack on the World Trade Centre (WTC) on 11 September 2001, as well as rescue workers; there was a surge in the incidence of sarcoidosis. Between 2002 and 2015, we found an age-adjusted incidence rate of 25 per 100,000 among male FDNY rescue/recovery workers [4].

Inorganic particles that are antigens, as well as various infectious agents, can enter the upper respiratory tract and lungs, triggering an autoimmune mechanism of inflammation. However, the inclusion of all factors in a multivariate model showed the significance of the effect of insecticides only [5,6].

The infectious etiology hypothesis of sarcoidosis has been proposed due to its resemblance to tuberculosis. Potential infectious agents associated with the development of sarcoidosis include *M. tuberculosis* and *Cutibacterium acnes* (formerly known as *Propionibacterium acnes*). Despite the detection of DNA residues by these microorganisms in sarcoid granulomas, no viable bacteria have been identified. Mycobacterial catalase-peroxidase, which serves as a target antigen for the secondary immune response and induces the formation of granulomatous inflammation, has been detected in the tissues of patients with sarcoidosis [5].

Sarcoidosis is a multisystem disease that can manifest as a wide range of symptoms at different time periods in different organs. Clinically, sarcoidosis can be categorized in different ways. For example, according to the type of onset, disease course, and organ involvement. The spectrum of clinical manifestations of sarcoidosis varies from the asymptomatic course to progressive and recurrent. There are also syndromes specific to it (Löfgren, Heerfordt syndromes) [6,7].

Sarcoidosis can be acute, subacute, or chronic, and in some cases can be asymptomatic. Patients with the acute form of the disease usually have a more positive prognosis associated with complete remission in the first two years [8,9]. Constitutional symptoms, such as fatigue, weight loss, and less frequent fever, are nonspecific and common to all forms of sarcoidosis. Patients usually present with marked fatigue, weight loss, and less frequently, fever, which are not nonspecific for any form of sarcoidosis [9].

Quite often, the disease may be asymptomatic. In some patients with acute-onset sarcoidosis, the disease is self-cured without long-term consequences, with complete remission within the first few years [10].

Given that the clinical manifestations of sarcoidosis are often nonspecific, histological examination of granulomas is often required to establish a diagnosis.

Obesity can also be considered a risk factor for sarcoidosis, as well as for other diseases whose pathogenesis is associated with alterations in the immune system (e.g., asthma and rheumatoid arthritis). In obesity, the immune cells that make up adipose tissue undergo a change in phenotype to a proinflammatory phenotype. These changes affect the lungs, and sarcoidosis, in particular, is characterized by chronic granulomatous inflammation, which is thought to be the result of a persistent immune response [8].

Owing to the great phenotypic diversity of the course of sarcoidosis, its diagnosis is still difficult. It is based on three main criteria: clinical presentation, histologic detection of non-caseating granulomas in one or more tissue samples, and exclusion of alternative granulomatous diseases [9,10].

It is assumed that sarcoidosis is an autoimmune disease, and the key link in its pathogenesis is the interaction of exogenous or endogenous antigens with components of innate immunity, such as macrophages and dendritic cells. However, the etiology of sarcoidosis remains poorly understood. It is initiated by one or more exposures to antigens that trigger the granulomatous process. Triggering factors that activate an inadequate immune response include infectious agents, vaccine components, and various inorganic substances.

Vimentin has been identified as one of the most likely endogenous antigens involved in the pathogenesis of sarcoidosis. Vimentin undergoes numerous modifications, potentially contributing to its immunogenicity. Elevated levels of antibodies have been obtained in sarcoidosis patients to modify citrullinated vimentin [6]. The immune response to vimentin can induce granuloma formation and cytokine responses in the lungs. However, given the presence of a humoral response to modify citrullinated vimentin in a number of autoimmune diseases, the presence of antibodies against vimentin in patients with sarcoidosis does not serve as a diagnostic marker for this pathology [7].

The histological features of sarcoid granulomas include the presence of well-defined, concentrically arranged layers of immunological cells. The core of the granuloma consists of centrally grouped macrophages and multinucleated giant cells that form the main layer. The peripheral layer consists of loosely organized lymphocytes, predominantly T-cells, which are often interspersed with a sparse population of dendritic cells. In some cases, granulomas are surrounded by isolated clusters of B-lymphocytes. Granulomas of sarcoidosis are usually characterized by a lack of necrosis; however, some variants of sarcoidosis may exhibit a heterogeneous mixture of necrotizing and non-necrotizing granulomas [11]. Although tissue histology may reveal an alternative diagnosis, granulomas do not have unique histological features in patients with sarcoidosis. It is necessary to differentiate these changes from other granulomatous diseases.

The lung is the organ most commonly affected by sarcoidosis; however, it is not the only target of this disease. Extrapulmonary sarcoidosis occurs in approximately 30–50% of patients [9].

Cutaneous manifestations, which are present in 15.9% [12] of patients with sarcoidosis, frequently remain undetected. This is primarily attributable to the diverse spectrum of cutaneous sarcoidosis presentations, encompassing erythema nodosum, maculo-papular lesions, areas of hyper- and hypopigmentation, keloids, and subcutaneous nodules [13]. Cutaneous lesions typically exhibit prominent regions of granulomatous inflammation, and the diagnosis can be established via skin biopsy.

Ocular involvement, which occurs in 11.8% [12] of patients, may precede the diagnosis of sarcoidosis by several years. The most frequently encountered manifestations include anterior uveitis, characterized by acute pain and visual disturbances, while photophobia is observed in a smaller proportion of cases [13].

Cardiac involvement in sarcoidosis is difficult to diagnose. Clinical symptoms are usually absent. If clinical symptoms and abnormalities are detected in functional studies, it is necessary to conduct an in-depth examination. The three criteria for cardiac sarcoidosis are conduction abnormalities, ventricular arrhythmias, and congestive heart failure. Granulomatous infiltration of the myocardium is the main cause of arrhythmias and cardiomyopathies [13].

Neurosarcoidosis is detected in 4.6% of cases [14]. Any part of the nervous system can be affected. The most common manifestations are cranial neuropathy and damage to the meninges. The II, VII, and VIII cranial nerves are affected. Meningitis can develop in one in five patients with neurosarcoidosis, with a significantly higher incidence of subclinical leptomeningeal lesions. Brain parenchymal disease is less common. Convulsions, headaches, and cognitive or behavioral disorders may be observed [15].

Sarcoid granulomas are also quite common in peripheral lymph nodes (11.3–15.2%), liver (4.5–11.5%), kidneys (3.1%), and skeletal-muscular systems (7.3%) [12,13,14].

The lack of clear diagnostic criteria for sarcoidosis requires differential diagnosis from other granulomatous diseases, where tuberculosis comes first. This fact must be taken into account in countries with a high burden of tuberculosis infection, which poses significant difficulties for correct diagnosis. Currently, according to the recognized criteria outlined in international recommendations, histological verification of the diagnosis is not exclusive, in contrast to previous views. Clinical, X-ray, CT, and immunological diagnoses must be included together.

We analyzed articles in PubMed, Scopus, Cochrane Library, and Embase, where databases were searched using the keywords “chronic sarcoidosis”, “diagnosis of sarcoidosis”, “course of sarcoidosis”, “pulmonary sarcoidosis”, “cardiac sarcoidosis”, “skin sarcoidosis”, “neurosarcoidosis”, “ocular sarcoidosis”, and “autoimmune inflammation”. Studies on the course and diagnosis of sarcoidosis with a deep search of ten years were included.

Objective of the study: To determine the course of different chronic sarcoidosis variants and markers for the identification of inflammatory activity.

## 2. Clinical Case of Chronic Sarcoidosis and Immunologic Features

Patient R., 33 years old, complained of a cough with sputum with blood streaks, weakness, and shortness of breath when walking. In December 2018, a routine fluorography revealed lymphadenopathy. After that, CT was performed, the results of which revealed focal shadows of low density “like frosted glass”; the foci merged into infiltrates of S6 of the right lung without clear contours with sizes 19 × 13 mm and 20 × 14 mm, in S3 of the left lung 11 × 11 mm, and in S2 of both lungs. Stage II sarcoidosis was suspected, but it was necessary to exclude disseminated tuberculosis as a priority; therefore, the patient was referred to the TB dispensary at the place of residence for additional examination.

The TB dispensary performed a test with the Diaskin test (negative), sputum microscopy, and luminescence microscopy twice (*M. tuberculosis*—not detected).

**Figure 1:**
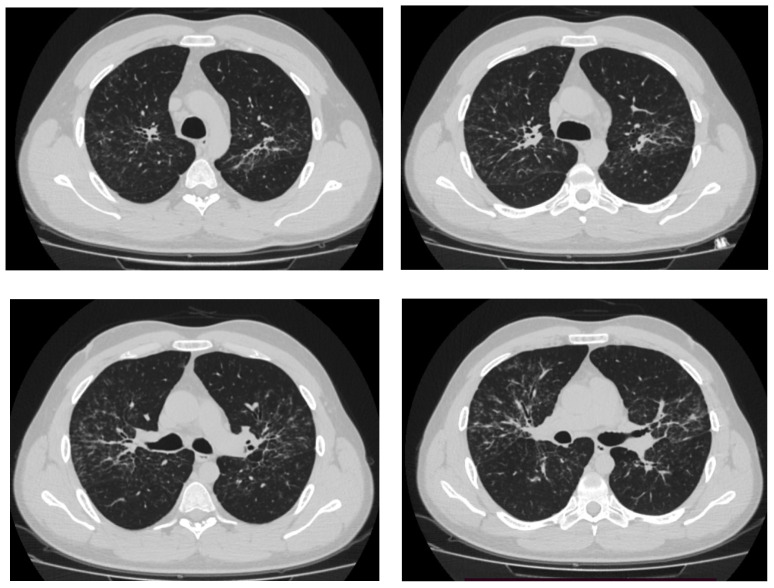
The results of MSCT of the chest organs.

According to the results of CT of the chest organs, expansion of the mediastinum and lung roots on both sides due to enlargement of lymph nodes was detected, and numerous foci and foci on the background of the enhanced pulmonary pattern with bullous swellings were detected in all lung sections (Figure 1). Then he was examined in the City TB Dispensary: T-SPOT (negative), fibrobronchoscopy with flushes, and transbronchial lung biopsy were performed. *M. tuberculosis* DNA was not detected in the flushes, and cultures were negative. Biopsy results revealed epithelioid granulomas without necrosis. The patient was referred to the sarcoidosis office of the City Hospital.

The patient was further hospitalized in a city hospital. CT of the chest from 17.09.2019 revealed polygonal foci with irregular contours along the bronchovascular bundles in the pericissural parts of the lung fields, merging into large infiltrates, corresponding to the picture of stage II sarcoidosis. The increase in intrathoracic lymph nodes became more pronounced: paratracheal and tracheobronchial up to 33 × 30 mm, to the left of the aortic arch and aortic window up to 24 × 15 mm, bifurcation up to 34 × 32 × 17 mm, bronchopulmonary on both sides up to 25 mm in the largest diameter, and supraclavicular up to 9 mm in the largest diameter. Fluid in the pleural cavities was not determined.

A significant increase in the volume and intensity of the changes in the dynamics was noted. When performing this study, an increase in the size of the liver and spleen was detected in the upper abdomen. After this finding, an ultrasound of the abdominal cavity was performed; lymphadenopathy of the abdominal cavity was detected—lymph nodes in the liver gates up to 30 mm in diameter and in the omentum—up to 14 mm in diameter.

Moreover, during hospitalization, bodiplethysmography was performed: OEL—within normal limits, in its structure: LEF within normal limits, with a tendency of OOL to decrease (within the conditional norm); lung air capacity at the level of quiet exhalation within normal limits, bronchial resistance during quiet breathing was not increased when assessing forced expiratory flow, and airway patency was within normal limits.

Fibrobronchoscopy with peritracheal biopsy of S3, S4, S5, and S8 of the right lung was repeatedly performed. Diffuse TB changes in the bronch, particularly on the right side, were observed. Repeated histologic examination of these masses showed numerous non-necrotic granulomas consisting of epithelioid and giant multinucleated cells in some places merging with each other without necrosis.

The patient was examined by an ophthalmologist, cardiologist, and otolaryngologist. No additional foci of granulomatosis were observed. The ACE level was 59.9 ACE units, with the norm of 20–70 ACE units. Since there were no data for tumor process and respiratory tuberculosis, the diagnosis of generalized sarcoidosis, histologically verified, was made according to the results of a complex examination. The patient was prescribed prednisolone at a dosage of 5 mg 4 times a day.

Against the background of the prescribed treatment, a positive dynamic was observed. Colleagues registered: cough decreased, general well-being improved, and hemoptysis did not recur. In 2020, he was hospitalized for a planned procedure for the examination and selection of therapy.

**Figure 2:**
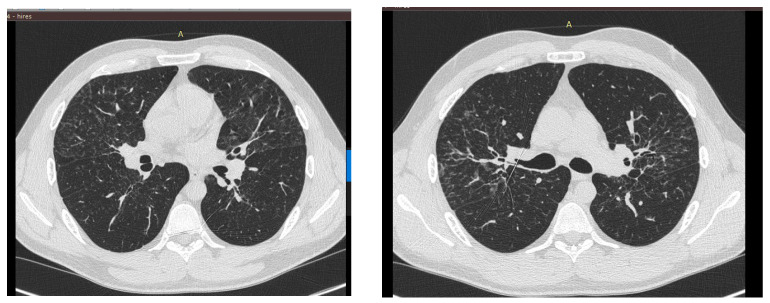
CT of the chest with positive dynamics.

Body plethysmography was performed, which showed an increase in flow indices up to normal limits with positive dynamics of volumetric and capacitive indices: an increase in OEL (+19%) due to an increase in OOL (+6%) and LEF (+22%). CT of the chest also showed positive dynamics of the disease course. CT of the chest also showed positive dynamics (Figure 2).

Ultrasonography of the abdominal cavity revealed positive dynamics: lymph nodes in the livser gates decreased to 20 mm, but hepatosplenomegaly persisted. A course of plasmapheresis with total exfusion of one DCP was carried out. It was recommended to continue taking prednisolone with a gradual decrease in dosage and repeat the course of plasmapheresis for 6–8 months.

Against the background of therapy in 2021, positive dynamics have continued to be observed. As a result of a CT scan of the abdominal cavity organs, the liver was reduced to a normal size, and the size of the spleen also decreased. However, pronounced lymphadenopathy persisted: prevascular lymph nodes 8.4 × 7.4 mm, para-aortic lymph nodes up to 11 × 8.1 mm, upper and lower paratracheal lymph nodes up to 14 × 8 mm, axial lymph nodes 17 × 10 mm. Based on the CT data, stage III sarcoidosis was diagnosed. The external respiratory function remained within normal limits. A course of plasmapheresis for correction and immunocorrection was carried out.

In 2022, the patient came for a follow-up appointment and complained of dizziness and recurrent cough. Ultrasound of the abdominal cavity organs again revealed splenomegaly enlargement of lymph nodes in the liver gates up to 12 mm in the largest diameter. On CT “fresh” focal and/or infiltrative character in the lung parenchyma was not found. However, there were signs of pulmonary fibrosis: compacted and irregularly thickened inter- and intra-lobular septa, focal thickening of linear-shaped areas of fibrous tenderness, and posterior segments of the upper lung lobes were slightly reduced in volume. The patient underwent an additional course of plasmapheresis.

In the control CT in 2023, no expression dynamics were observed. On ultrasound remains, splenomegaly and lymphadenopathy (enlarged lymph node 18.5 × 7.3 mm in the liver gate without dynamics).

In 2023, against the background of clinical well-being, a complex, in-depth immunological examination was performed (Table 1).

According to the data presented in Table 1, there is an increase in the level of B-cell total (CD3-CD19+)–29.87, as well as an increase in the absolute values of T-cells, which reflect the presence of an active infectious and autoimmune inflammatory process.

In the control CT in 2023, no pronounced dynamics were observed. According to ultrasound, splenomegaly and lymphadenopathy (enlarged lymph node 18.5 × 7.3 mm in the liver gates without dynamics) persisted, which indicated the progression of the process. The examination data correlated well with the results of the immunologic changes. The results obtained could be an indication for the prescription of immunotherapy with leflunomide.

## 3. The Potential Manifestations of Acute Sarcoidosis

Löfgren’s syndrome is a clinical variant of sarcoidosis that reflects its acute debut and presents as a triad that includes bilateral intrathoracic lymphadenopathy, fever, erythema nodosum, and/or migratory polyarthritis (predominantly in the ankles, but knees or wrists may be affected). Acute-onset results in erythematous and painful nodules that are usually located on the bilateral extensor surfaces of the lower extremities. The appearance of nodules may be preceded by a prodromal period (7–21 days), manifested by fatigue, fever, malaise, arthralgia, or symptoms of upper respiratory tract infection [16,17]. The symptom complex reaches its maximum in 1 to 2 weeks, and the time to remission is usually 3 to 12 months. Nodular erythema and fever usually recover spontaneously within 1 to 6 weeks, whereas resolution of lymphadenopathy may take a year or more [18].

Another syndrome characteristic of the acute debut of sarcoidosis is the Heerfordt-Waldenström syndrome (uveoparotid fever). This syndrome is extremely rare in clinical practice (5 to 10%) but unambiguously indicates the presence of sarcoidosis in the patient [19]. As a rule, patients with the complete form of the disease are diagnosed with four symptoms: uveitis, enlargement of the parotid salivary glands, subfebrile fever, and facial nerve palsy (Bell’s palsy); with the incomplete form, two or three symptoms are present. Parotid gland involvement is observed in 65% of cases as recurrent diffuse painless swelling, often with xerostomia. Sometimes, it is bilateral and can reach the submandibular and lacrimal glands. Facial nerve paralysis is inconsistent and is seen in 25–50% of cases [20]. The diagnosis of Herefordt-Waldenström syndrome is made on the basis of the clinical picture and lymph node biopsy revealing non-caseating granulomas.

## 4. Different Manifestations and Causes of Pulmonary and Intrathoracic Lymph Node Sarcoidosis

In sarcoidosis, pulmonary involvement is observed in the majority of cases, and mediastinal lymphadenopathy is nearly diagnosed. Clinical manifestations exhibit a wide spectrum, ranging from an asymptomatic course detected incidentally on chest radiography to progressive and culminating in respiratory failure. Respiratory symptoms are nonspecific, with dyspnea and cough being the most frequently reported, while wheezing and chest pain may also be presented [21].

The development of sarcoidosis can proceed along three main pathways. The asymptomatic course of the disease, which we mentioned earlier. The second pathway is characterized by a non-fibrotic symptomatic course, in which patients present with pulmonary symptoms, intraparenchymal granulomatous inflammation on chest imaging, and a mild decrease in pulmonary function. Progressive deterioration is seldom observed in these patients. However, the disease may persist for months, years, or even a lifetime and may necessitate immunosuppressive therapy. The third trajectory is a progressive fibrotic disease, which afflicts 10–20% [22] of patients with sarcoidosis.

The vast majority of deaths from sarcoidosis occur in this subgroup of patients because of complications of interstitial lung disease, including bronchiectasis leading to severe lung infection, pulmonary hypertension, and pulmonary aspergillosis leading to hemoptysis. Pulmonary hypertension develops not only due to pulmonary fibrosis and the resulting destruction of parenchyma and capillary occlusion, but also due to extrapulmonary pathologies such as cardiac, vascular, and hepatic involvement. For this reason, pulmonary hypertension in sarcoidosis develops with complex factors [23].

Pulmonary aspergillosis is caused by the growth of *Aspergillus spp.* into multiple cysts that form during the development of pulmonary fibrosis in the upper lobes. This condition tends to become refractory to antifungal therapy and may be complicated by pneumothorax [24].

Chest radiography and computed tomography are key investigations in pulmonary sarcoidosis [25].

Staging is essential for prognosis and does not reflect the natural course of pulmonary sarcoidosis. As the radiological stage advances, the probability of spontaneous remission diminishes without treatment [26]. This system remains in use for the clinical classification of sarcoidosis patients, research, and for providing prognostic information to patients, partly due to its simplicity. The ubiquity, cost-effectiveness, and low radiation exposure associated with chest radiography have also contributed to the widespread utilization of the Scadding system.

CT is the optimal modality for the initial evaluation of patients with suspected or confirmed sarcoidosis, as it enables the assessment of the lungs and airways with significantly higher resolution in multiple planes and exhibits superior sensitivity and specificity. CT characteristic findings in a patient with sarcoidosis include bilateral enlargement of mediastinal lymph nodes, nodules with a perilymphatic distribution along the interlobular septa, subpleural regions, and bronchial vessels, which may consolidate into extensive opacities. Sarcoidosis granulomas are most frequently situated along the perilymphatic areas and branches of the bronchial tree [27]. Airway involvement can be macroscopically observed during bronchoscopy; the bronchial mucosa appears erythematous and cobblestone in appearance, and biopsy of these sites corroborates the presence of non-caseating granulomas [28].

The identification of non-caseating granulomas in at least one biopsy is a prerequisite for confirming the diagnosis. However, this is not necessary when there is a definite clinical picture, such as in Löfgren’s or Heerfordt’s syndromes. In most cases, it is sufficient to detect granulomas in only one biopsy. A second biopsy may be justified in cases of possible visceral manifestations that are difficult to relate with certainty to sarcoidosis [29].

When performing biopsies, less invasive sites should be prioritized (e.g., skin lesions if present in association with lung lesions). In the absence of superficial lesions, it is recommended that samples be obtained during bronchial endoscopy. In rare cases, CT-guided transthoracic lung biopsy is required [30].

## 5. Difficulties in the Diagnosis of Cardiac Sarcoidosis

Cardiac sarcoidosis is a significant and fatal manifestation of extrapulmonary sarcoidosis. The formation of non-caseating granulomas is progressive and causes inflammation in all three layers of the heart. The most common localization of non-caseating granulomas is the myocardium.

The detection rate of cardiac sarcoidosis has increased over the past 25 years due to improved diagnosis, including imaging techniques [31]. Approximately 5% of patients with sarcoidosis have clinical manifestations of cardiac involvement. In this case, cardiac clinical manifestations prevail over extracardiac manifestations; however, in 20% of patients, the disease is asymptomatic [32].

Cardiac manifestations depend on the localization and degree of disease activity. These can range from an asymptomatic course to ventricular tachycardia, atrioventricular block, heart failure, or sudden cardiac death. Supraventricular arrhythmias are less common. The diagnosis of cardiac sarcoidosis presents clinical challenges due to phenotypic and histologic overlap with other inflammatory heart diseases. Diagnosis should be made in patients with extracardiac sarcoidosis, regardless of the presence of clinical manifestations. Two criteria must be met to make the diagnosis of cardiac sarcoidosis: histologic and clinical criteria. The histological criteria include the finding of non-caseating epithelioid granulomas in an endomyocardial biopsy or other surgical specimens [33]. The use of cardiac magnetic resonance imaging in the early treatment of chronic inflammatory heart disease is increasing. However, quantitative mapping is currently gaining a special role in the monitoring and treatment of sarcoidosis [34].

The diagnosis is also possible if epithelioid granulomas are present in other organs and two or more major criteria or one major and two or more minor criteria listed below.

*Main criteria:*

- High-grade atrioventricular block or fatal ventricular arrhythmia;
- Basal thinning of the interventricular septum or abnormal ventricular wall anatomy;
- Abnormally high 67Ga citrate or 18F-FDG-PET uptake;
- Decreased left ventricular ejection fraction less than 50%;
- Delayed improvement on gadolinium-enhanced MRI.

*Minor Criteria:*

- Ventricular arrhythmias registered on ECG, deviation of the electrical axis of the heart, or abnormal Q teeth;
- Perfusion defects on myocardial perfusion scintigraphy data;
- Monocytic infiltration and moderate or severe interstitial fibrosis of the myocardium according to endomyocardial biopsy.

The electrocardiogram is usually abnormal in patients with clinical symptoms of cardiac sarcoidosis. Abnormalities include conduction blocks of varying degrees, such as isolated bundle branch blocks, abnormal Q (pseudoinfarction pattern) ST-T changes, and, rarely, epsilon waves. In asymptomatic patients, these abnormalities are found in only 3.2–8.6% [32].

Triggers such as complaints of palpitations, syncope, atypical chest pain, dyspnea, decreased exercise tolerance, physical examination findings (ballooning of jugular veins, heart auscultation murmurs, gallop rhythm), or unexplained heart failure should prompt the clinician to initiate an evaluation for cardiac sarcoidosis using continuous ambulatory ECG (Holter) and echocardiogram [33].

The diagnostic difficulties lie in the fact that endomyocardial biopsy has low sensitivity due to the heterogeneous distribution of granulomatous inflammation. Pathognomonic granulomas with multinucleated giant cells can be detected in only 20% of biopsy specimens [34], which is due to the uneven distribution of granulomas that can involve any layer. The myocardial area occupied by granulomatous lesions is the left ventricular free wall [35].

At the same time, imaging techniques are not specific enough to accurately establish a diagnosis. Laboratory tests are also not pathognomonic and may include anemia, decreased white blood cell count, or increased erythrocyte sedimentation rate [35].

Transthoracic echocardiography is the least sensitive tool for the diagnosis of cardiac sarcoidosis; however, it is specific, and the abnormalities detected may be useful for detecting cardiac dysfunction in the presence of extracardiac sarcoid granulomas. Moreover, since left ventricular ejection fraction is one of the most important prognostic indicators, once diagnosed, this relatively inexpensive imaging method plays a role in the management of patients with cardiac sarcoidosis [31]. Prospective studies suggest that new indices, such as global longitudinal strain, may have an advantage over conventional transthoracic echocardiography in providing diagnostic and prognostic information [35].

Cardiac magnetic resonance imaging is one of the most important tools for the diagnosis of cardiac sarcoidosis, mainly by detecting late gadolinium enhancement in the myocardium with a characteristic pattern of patchy and multifocal uptake with preservation of the endocardial border. Several studies have shown an association between late gadolinium enhancement and poor prognosis [36].

Positron emission tomography with 18-fluorodeoxyglucose is most useful for visualizing sites of myocardial inflammation as well as monitoring the response to immunosuppressive therapy. The presence of 18-fluorodeoxyglucose uptake and perfusion defects on PET are associated with a higher risk of cardiac death or ventricular tachycardia. In particular, patients with focal right ventricular inflammation had a 5-fold higher event rate compared with patients with normal perfusion and metabolism, suggesting that a focal right ventricular lesion may be a marker of more severe disease [37].

Advanced cardiac imaging includes cardiovascular magnetic resonance imaging and 18-fluorodeoxyglucose-positron emission tomography.

*The indications for performing advanced imaging are as follows:*

- Patients with verified extracardiac sarcoidosis who met one of the following criteria:
- One or more of the following symptoms: significant palpitations of more than 1–2 weeks duration, history of pre-syncope or syncope.
- One or more of the following ECG abnormalities: complete left or right bundle branch block; presence of unexplained abnormal Q-squares in two or more leads; persistent first, second, or third-degree atrioventricular block; and persistent or non-persistent ventricular tachycardia.
- One or more of the following echocardiographic abnormalities: regional wall motion abnormalities, ventricular aneurysm, basal septal thinning, or left ventricular ejection fraction less than 50%.

Patients without extracardiac sarcoidosis and those with one or more of the following criteria:

- Unexplained second- or third-degree Mobitz II or third-degree atrioventricular block in adults less than 60 years.
- Sustained monomorphic ventricular tachycardia in the absence of any known etiology [38].

Patients with extracardiac sarcoidosis without current signs and symptoms of cardiac sarcoidosis should be prospectively followed up with serial (e.g., annual) clinical examinations and ECGs to monitor the possible development of signs and symptoms.

The diagnosis of cardiac sarcoidosis can be definite and indeterminate. Definite cardiac sarcoidosis is established when a non-caseating granuloma is found on histological examination of the myocardial tissue without identifying an alternative cause [35]. Since histologic findings are not pathognomonic, some experts consider the diagnosis of cardiac sarcoidosis to be “highly probable” when non-caseous granulomas are found.

Uncertain cardiac sarcoidosis includes the categories of highly probable, probable, and possible. The term may be used when there is uncertainty regarding the diagnosis [38].

Patients with clinically manifested cardiac sarcoidosis have a 10% risk of sudden cardiac death during 5 years of follow-up. During the subclinical course, the risk is unknown. One of the direct complications of cardiac sarcoidosis is ventricular arrhythmia, which is a strong predictor of mortality [39].

## 6. Diagnostic Possibilities of Skin Sarcoidosis

The cutaneous system is the second most frequently affected organ system in sarcoidosis [40]. Cutaneous sarcoidosis manifests in one-third of patients with systemic sarcoidosis; less commonly, it can also present as an isolated finding. Notably, cutaneous sarcoidosis is frequently associated with additional systemic involvement, particularly in young female patients [41]. Sarcoidosis-related skin lesions can develop on any cutaneous surface, but they are more pronounced in previously traumatized areas, such as tattoos and scars [42]. Manifestations can be classified as either specific or nonspecific. In nonspecific lesions, granulomas are microscopically absent. The clinical presentation may vary depending on the morphological characteristics of the lesion, chronicity, and the patient’s skin pigmentation [43].

Among the specific manifestations of sarcoidosis, the following are the most common:

- Papules and papulovesicles: numerous, less than 1 cm in size, firm, and usually scaly. Color may be flesh-colored, yellow-brown, purplish-brown, or hypopigmented. Inflammations are usually located on the face, less commonly on the trunk and extremities, and may occur in scars.
- Plaques: oval or annular in shape, often well-circumscribed, usually firm to touch, sometimes scaly. Color varies from red-brown to flesh-colored, purplish-brown, and sometimes yellow-brown. They are located on the trunk, buttocks, shoulders, forearms, and lower back.
- Chilling lupus erythematosus: smooth, shiny plaques that are brown to purple or erythematous and may have scales on them. Inflammation occurs in the central part of the face, especially in the nose, cheeks, lips, forehead, and ears.
- Subcutaneous nodules are dense, mobile, rounded, or oval and are erythematous, flesh-colored, purple, or hyperpigmented. Inflammation is located on the extremities, predominantly the upper extremities, and less frequently on the trunk [40].

The less common forms include the following:

- Ichthyosiform (polygonal dense scales of brown or white-gray color);
- Atrophic and ulcerative;
- Sarcoidosis of the mucous membranes (cheeks, gingiva, hard palate, tongue, back of the pharynx, and salivary glands are involved, with papules, plaques, nodules, or infiltrative thickening);
- Erythroderma;
- Erythroderma (papules, plaques, nodules, or infiltrative thickening);
- Erythroderma (coalescence of thickened, yellow-brown, red-brown, or purplish-brown scaly plaques covering extensive cutaneous areas, often accompanied by small superficial scales or mild exfoliative dermatitis);
- Alopecia (scarring or non-scarring);
- Nail sarcoidosis (thinning, brittleness, thickening, pitting, furrows, trachyonychia, hyperpigmentation, nail plate destruction, and drumstick symptoms) [40].

The most common clinical manifestation is lupus pernio, which disproportionately affects African Americans and females. It exhibits a chronic and refractory course that frequently requires aggressive systemic therapy. The distinctive appearance of lupus pernio facilitates its recognition as a manifestation of sarcoidosis. However, certain differential diagnoses may mimic its clinical presentation, including fungal infections, discoid lupus erythematosus, berylliosis, cutaneous lymphoma, systemic lupus erythematosus, and tuberculoid leprosy, which exhibit a similar pattern of dissemination [44].

Diagnosis of the cutaneous form is rarely difficult because the skin is an easily accessible organ for biopsy. Among the specific manifestations of skin sarcoidosis, non-caseous epithelioid granulomas are found in the biopsy specimens. However, it should be remembered that the cutaneous form is most often one of the manifestations of generalized sarcoidosis; therefore, the diagnostic search should be directed to the detection of granulomas in other organs.

## 7. The Potential Manifestations and Diagnostic Criteria of Sarcoidosis of the Nervous System

Nervous system involvement in sarcoidosis accounts for 5–10% of patients and up to 26% of patients with generalized sarcoidosis [45] and can lead to potentially dangerous consequences. Nervous system inflammation develops within the next 2 years in approximately 75% of patients after the diagnosis of sarcoidosis [46]. Isolated neurosarcoidosis was previously thought to be rare, but recent studies have shown that of all cases diagnosed with sarcoidosis, 10–20% are isolated nervous system lesions [47].

The common clinical manifestations of neurosarcoidosis can be divided into those affecting the central nervous system (brain, spinal cord, and cranial nerves) and those affecting the peripheral nervous system (peripheral nerves). We can refer to these manifestations as cranial neuropathies, myelitis, and intraparenchymal lesions. In general, severe neurological symptoms are rarely diagnosed. At the beginning, there may be neuropsychiatric disorders in the form of depression (60–66% of cases) and psoriasis (20% of cases). More often, these manifestations may occur in people with neurosis [46] and encephalopathy [48]. These results have been reported previously.

It should be noted that central nervous system (CNS) damage in sarcoidosis results from the spread of leptomeningeal inflammation through the perivascular spaces, which are most pronounced and numerous at the base of the brain and provide communication between the lymphatic fluid and parenchyma [49]. Neurosarcoidosis most often manifests as cranial neuropathy in 50% of the cases. The facial nerve and optic nerve are affected. Hearing loss, neuritis, and sensory disorders are quite common [47].

Granulomatous infiltration of the nuclei, bundles, or cranial nerves causes cranial neuropathy. Multiple simultaneous or sequential cranial neuropathies should raise the clinician’s suspicion of neurosarcoidosis. The disease course is usually subacute and progressive. Most patients have additional neurological symptoms [46].

Optic neuritis or optic perineuritis may involve the optic nerve junction or cause compression optic neuropathy due to infiltration or tumor involvement. In neurosarcoidosis, optic neuritis is more often bilateral and the prospects for visual recovery are limited [46].

Recurrent bilateral facial nerve palsy (simultaneous or sequential) or other nearby cranial neuropathies should raise a clinician’s suspicion of neurosarcoidosis. Herfordt’s syndrome is a rare manifestation of neurosarcoidosis [46].

Aseptic meningitis is usually characterized by leptomeningeal inflammation and less commonly by pachymeningeal inflammation. It usually manifests as headache and cranial neuropathy. Occasionally, seizures, cognitive impairment, and gait disturbances are observed [50]. In severe cases, the disease may be complicated by hydrocephalus with dulling of consciousness, requiring urgent evaluation and possibly shunt placement if a patient does not respond to medical treatment [49].

Intraparenchymal brain lesions may present with or without involvement of the dura mater, cognitive changes, gait disturbances, and focal sensorimotor disorders, depending on the localization of the lesion. The involvement of basal neuroendocrine structures can lead to hypothalamic-pituitary dysfunction, which is often irreversible. Anterior hypopituitarism (LH/FSH 89%, TSH 67%, GH 50%, ACTH 49%), hyperprolactinemia (49%), and diabetes mellitus (65%) [46] are reported quite frequently. Stroke is rare and may be due to perivascular inflammation with invasion of the vessel wall, vasculitis (rare), or direct granulomatous compression of the intracranial artery [49].

Spinal cord involvement represents one of the most severe clinical manifestations, as it can frequently result in residual paraparesis, sensory dysesthesia, and pelvic organ dysfunction [49]. Sarcoidosis can affect the spinal cord through various mechanisms, including infiltration of the parenchyma, leptomeningeal structures, extradural space, and extraspinal tissues, with compression of the spinal cord. Contemporary studies have proven the presence of myelopathy in approximately 19–26% of patients, and it is one of the most common neurological manifestations of neurosarcoidosis [46].

Sarcoidosis of the peripheral nervous system is manifested by the appearance of polyneuropathy of both large and small nerve fibers or polyradiculoneuropathy with purely motor, sensory, or mixed sensorimotor features. Demyelinating polyneuropathy may also occur. It is similar to chronic inflammatory demyelinating polyneuropathy and Guillain-Barré syndrome [46]. Large myelinated peripheral nerve fibers are rarely affected and account for less than 2% of all neurological complications. Sarcoidosis myopathy usually presents with gradually progressive weakness in a proximal-distal direction, with rare involvement of the neck, face, bulbar, and respiratory muscles. Less common muscular manifestations include nodular myopathy, acute myositis, and predominant distal involvement resembling inclusion myositis [49]. Other manifestations include multiple mononeuritis, pure sensory or pure motor neuropathy, and impingement syndromes [50].

In addition to neurological granulomatous manifestations, patients with sarcoidosis experience small fiber neuropathy and headaches that are not granulomatous in nature. The etiology of these manifestations is poorly understood and could potentially be related to a number of factors, including active inflammation, cytokine release, and side effects of drug therapy [49,50,51].

The highest level of diagnostic certainty for neurosarcoidosis is attained through pathoanatomical verification; however, in certain cases, neuro-anatomical localization may preclude biopsy due to concerns regarding morbidity or the possibility of establishing the diagnosis through less invasive methods [46].

A biopsy is necessary to diagnose definite (nervous system biopsy) and probable (biopsy of systemic foci) neurosarcoidosis. However, this may not be sufficient to make a diagnosis, as there are many granulomatous diseases, including infections, autoimmune conditions, and neoplastic diseases. It may mimic sarcoidosis and initially respond to corticosteroid treatment. Similar granulomatous diseases may mimic sarcoidosis and initially respond to corticosteroid treatment. Patients with a biopsychologically confirmed diagnosis of systemic sarcoidosis tend to overlook other etiologies when they develop cranial neuropathies or leptomeningeal disease suggestive of neurosarcoidosis [48].

For patients with suspected neurosarcoidosis, magnetic resonance imaging (MRI) and cerebrospinal fluid (CSF) examination may be beneficial for detecting inflammation. In patients without a history of sarcoidosis (50% of cases), histological confirmation of the affected part of the nervous system or an extra-neural source that may also be involved should be performed. For patients with known systemic sarcoidosis, histologic confirmation of the affected nervous tissue is less critical, but every effort should be made to exclude diseases that present with similar clinical and imaging findings [49].

Specifically, neurosarcoidosis should be differentiated from all granulomatous infections, including tuberculosis, syphilis, Whipple’s disease, brucellosis, nocardiosis, actinomycosis, fungal lesions (aspergillosis, cryptococcosis, endemic mycoses), and parasitic lesions (toxoplasmosis, schistosomiasis, neurocysticercosis, and toxocarosis). This step is mandatory before the administration of immunosuppressive therapy.

Radiological diagnostic techniques remain the mainstay for the evaluation of changes in sarcoidosis. MRI with gadolinium is the most important diagnostic modality for the evaluation of CNS sarcoidosis. It is important to realize that a wide range of changes can be seen in this study, which may include nonspecific periventricular white matter lesions, enhancement/thickening of cranial nerves or basal neuroendocrine structures, and intraparenchymal masses with gadolinium enhancement. The leptomeningeal enhancement with a predominant basilar component is quite often noted, and on MRI, it often has the appearance of mottled nodules. Patients with spinal cord lesions may also exhibit focal and nodular meningeal enhancement, which helps distinguish it from multiple sclerosis [49].

Depending on the affected area of the brain, lesions in sarcoidosis can be hypo- or hypermetabolic. Occasionally, spinal cord lesions manifest hypermetabolic abnormalities since the basic metabolism of the spinal cord is one-third of that of the brain gray matter. Metabolic changes allow FDG-PET to determine the dynamics against the background of the therapy before morphological changes appear [52].

Cerebrospinal fluid analysis can help establish the presence of inflammation and exclude other possible diseases. However, there are no changes specific to neurosarcoidosis that are characteristic of CNS sarcoidosis. A moderate pleocytosis (usually <100 cells/μL) with a predominance of lymphocytes and increased protein content is noted; neutrophils may also be present, especially in the acute form; and eosinophils in CSF are rare. Isolated protein elevation in the CSF may be a marker of inflammation, but is nonspecific, especially in the absence of MRI evidence of CNS sarcoidosis [46]. An elevated CSF glucose level of 40 mg/dl or a low level of less than 40% depends on serum glucose levels and occurs in 25–31% of cases. However, these changes are also nonspecific. Oligoclonal bands and elevated IgG values may be detected [49]. In about 15% of patients, changes in the CSF may be absent [52]. CSF cytology and flow cytometry should be considered to assess for malignancy. CSF studies may also be useful for monitoring neurosarcoidosis disease activity over time, such as in response to treatment, especially opening pressure, cell count, total protein, glucose, IgG index, and oligoclonal bands [46].

## 8. Difficulties in the Diagnosis of Ocular Lesions in Sarcoidosis

Ocular sarcoidosis is the second most commonly affected extrapulmonary sarcoidosis, with the isolated form in 7.7–32.9% of cases [53].

Sarcoidosis can affect any structure of the eye as well as its appendages. However, uveitis (anterior, intermediate, and posterior) remains the most frequent manifestation (up to 30%) and is the most threatening manifestation, as it can lead to vision loss. Conjunctival granulomas are also quite common and, in most cases, asymptomatic. Other ophthalmologic manifestations of sarcoidosis include dacryoadenitis, orbital inflammation, eyelid granuloma, madarosis, polyosis, and keratitis [54].

Anterior uveitis can manifest as either acute (sudden onset and duration <3 months) or chronic (recurrence within 3 months of discontinuing treatment), with the latter being more prevalent. In anterior uveitis, the primary focus of inflammation is the anterior chamber, which is defined as iritis, iridocyclitis, or anterior cyclitis. Classical anterior uveitis in ocular sarcoidosis is bilateral and granulomatous, with anterior and posterior synechiae (adhesions between the iris and cornea and between the iris and lens, respectively). Intraocular hypertension may also be observed [53].

Granulomatous uveitis presents as large keratinous “mutton fat” precipitates or iris nodules located either at the pupillary margin (Koeppe’s nodules) or within the iris stroma (Busacca’s nodules) [55,56].

In intermediate uveitis, the primary focus of inflammation is the vitreous body. It is not frequently encountered in patients with sarcoidosis (6–19%) [53]. The most common features of intermediate uveitis in sarcoidosis are “snowballs” in the vitreous, which may be organized into a “string of pearls” configuration [56]. The primary cause of vision loss in patients with intermediate uveitis is cystoid macular edema, followed by vitreous opacity, epiretinal membrane formation, optic neuritis, and glaucoma [53].

The retinal or vasculature of the eye is affected by posterior uveitis, which is the main focus of inflammation in sarcoidosis. During the diagnostic search, choroiditis, chorioretinitis, retinochorioiditis, retinitis, and neuroretinitis should be defined. Posterior uveitis is observed in 5–28% of sarcoidosis cases with ocular involvement [53]. Peripheral and central multifocal choroiditis are the most common characteristics of sarcoidosis. Choroidal granulomas are less common but are highly characteristic of sarcoidosis. They are larger than the foci of multifocal choroiditis and may be located around the optic disc or in the peripheral retina [56]. Sarcoidosis is a major cause of panuveitis (a combination of inflammation in the anterior chamber, vitreous, and retina or vasculature) and accounts for up to 48% of all uveitis in sarcoidosis [53].

Retinal vasculitis is found in 18% of patients with ocular sarcoidosis [53]. It often accompanies intermediate or posterior uveitis, as well as panuveitis. It most often presents as periphlebitis, which may involve capillaries. The most classic finding is “candle wax drops” in perivenous retinal sheaths and infiltrates [56]. Occlusive vasculitis (mostly venous) and ischemia are rare, but they are classic manifestations that can lead to retinal neovascularization in 1–5% of cases [53].

In ocular sarcoidosis, multimodal imaging plays a crucial role in the diagnosis of important clinical features. Optical coherence tomography (EDI-OCT) can determine the presence of macular edema and choroidal granulomas. Fluorescein angiography is important for the diagnosis of retinal vasculitis and optic disc edema. In cases of retinal vasculitis, fluorescein angiography is mandatory to detect retinal occlusive vasculitis and retinal ischemia and to determine the need for retinal photocoagulation. Angiography with indocyanine green is useful for studying choroidal lesions, such as choroidal granulomas or choroiditis [53].

The diagnosis of ocular sarcoidosis cannot be established solely on the basis of ophthalmological findings. Histopathologic confirmation of non-caseating granulomas in biopsy specimens is considered the gold standard for diagnosis. However, biopsy of intraocular tissues, such as the iris, retina, or vasculature, is rarely performed due to the potential risk of irreversible damage to these tissues, which could lead to visual dysfunction [57]. Therefore, it is ideal to utilize the results of systemic studies, the diagnostic search of which is aimed at identifying the foci of sarcoidosis in other organs.

## 9. Immunology Futures in Chronic Sarcoidosis

As noted earlier, the diagnosis of sarcoidosis requires three criteria: appropriate clinical and radiological findings, histological verification of non-caseating granulomatous inflammation, and exclusion of alternative granulomatous diseases that may present with similar clinical and histological features. Thus, histologic, clinical, and radiologic findings alone are insufficient to make a diagnosis, making sarcoidosis a diagnosis of exclusion.

However, sarcoidosis is an important diagnosis that must be established because timely initiation of appropriate treatment can not only help improve the quality of life of the patient but also avoid lethal outcomes. At the same time, misdiagnosis of sarcoidosis can lead to inadequate treatment of alternative diseases, which can have serious and life-threatening consequences.

As with any other disease, the diagnostic search began with the collection of clinical data, including complaints, examination, history of the disease, and life history. Based on these findings, the physician must assess the likelihood of sarcoidosis. If the probability estimate, in the opinion of the physician, is extremely low, the diagnosis of sarcoidosis is not further considered. If the doctor sets the probability of diagnosis, additional laboratory and instrumental diagnostic methods are prescribed, including histological examination of the tissue suspected to be affected by the granulomatous process.

Alternative granulomatous diseases that should be excluded include infections (particularly tuberculosis, nontuberculous mycobacterial infections, and histoplasmosis), chronic berylliosis, hypersensitivity pneumonitis, granulomatous talcosis, drug-induced granulomatosis (especially due to the administration of TNF-α antagonists, immune checkpoint inhibitors, targeted therapies, and interferons), immunodeficiency, genetic disorders (Blau syndrome), Crohn’s disease, granulomatosis with polyangiitis, eosinophilic granulomatosis with polyangiitis, and malignancy-associated granulomatosis. Excluding lymphoproliferative diseases can also be exceptionally challenging until a characteristic biopsy specimen is obtained.

In sarcoidosis, epithelioid cell granulomas are well-formed structures whose compact core consists of macrophages and cells originating from macrophages (epithelioid and giant cells) closely associated with CD4+ T-lymphocytes. The peripheral component contains CD8+ lymphocytes, CD4+ FOXP3+ Treg cells, Th17 cells, B-lymphocytes, and IgA-producing plasma cells. A characteristic feature that is common but not specific to sarcoidosis is the presence of cytoplasmic inclusions, primarily within multinucleated giant cells [58]. Notably, sarcoid granulomas are particularly prominent and tend to be confluent. Granuloma clusters can form macroscopically visible but small white nodules (micronodules) or large masses (macronodules), with relative preservation of the intervening tissue [22].

Although there is no biomarker specific for sarcoidosis, polyclonal hypergammaglobulinemia is often seen in this disease, but never low gammaglobulin levels on serum protein electrophoresis. Moreover, when sarcoidosis is suspected, hypercalcemia with low parathyroid hormone levels and normal or low 25-hydroxycalciferol levels may be favored [29].

Some studies have also indicated the significance of serum angiotensin-converting enzyme (ACE) levels in the diagnosis and determination of activity status. ACE can be secreted by monocytes, macrophages, and epithelioid cells and is involved in the pathogenesis of sarcoidosis as an important modulator of granuloma formation and correlates with the presence of granulomas and radiologic stages II and III [59,60].

However, Zhou, Y. et al., in their study [61], found a relationship between elevated sAPP levels and multiorgan involvement. ACE levels were increased in patients with lesions in the extrathoracic lymph nodes, skin, and spleen, along with abnormal calcium metabolism.

Another marker hypothesized to correlate with disease activity is the serum-soluble interleukin-2 receptor (sIL-2R). Upon activation, Th1 cells increase IL-2R expression on the cell surface and release sIL-2R into the bloodstream. Thus, elevated levels of sIL-2R are considered a marker of Th1 cell activation during granuloma formation and maintenance [59]. Increased levels of this marker are not specific to sarcoidosis, as it is also found in other granulomatous diseases, hematologic malignancies, and various autoimmune diseases. However, studies have shown that sIL-2R levels can be used to predict disease progression or relapse after discontinuation of therapy [59].

Since the clinical manifestations of sarcoidosis are quite diverse, as described above, various instrumental methods are used to diagnose sarcoidosis.

In pulmonary sarcoidosis, chest radiography is most commonly used, which reveals abnormalities in most cases. In addition, on the basis of this examination, the Scadding classification operates, which not only allows us to stage the disease but also stratify the risk of an unfavorable outcome. However, chest radiography has proven to be an unreliable predictor of pulmonary function abnormalities on initial evaluation; it has a lower sensitivity for parenchymal disease compared with high-resolution computed tomography. Computed tomography has a higher diagnostic accuracy because it can detect the smallest parenchymal lesion that is not apparent on conventional radiographs. Numerous studies have demonstrated a significant frequency of parenchymal changes on CT scans in patients with Scadding stage 0 or I [26].

Additionally, all patients with established or suspected pulmonary sarcoidosis should undergo an external respiratory function test. Functional test results usually correlate with the overall disease process but do not always reflect the radiologic stage. Restriction of lung volume, especially the forced vital capacity of the lungs (FVCL), is the most common finding on spirometry. The ratio of forced expiratory volume in one second (FEV1)/FGEF may be reduced in cases of significant bronchial deformity and stenosis due to pulmonary fibrosis, diffuse bronchial granulomatosis, proximal endobronchial stenosis, bronchial compression due to lymphadenopathy, granulomatous bronchiolitis, or bronchial hyperreactivity [56].

The diagnosis of sarcoidosis is often confirmed using bronchoalveolar lavage fluid (BALF) obtained during bronchoscopy. The percentage of lymphocytes often increases in patients with sarcoidosis. Lymphocytosis > 15% is particularly important, as is an increase in the CD4/CD8 T-cell ratio of 3.5 or more [59]. However, these parameters are not specific to sarcoidosis, do not reflect the severity of the disease, and can be used in conjunction with other methods of investigation.

When cardiac involvement is suspected, transthoracic echocardiography is the initial imaging modality because it is the most accessible and noninvasive method. Commonly described echocardiographic findings in sarcoidosis include abnormalities in regional wall motion, aneurysms, basal septal thinning, left ventricular dilatation, and impaired right or left ventricular systolic or diastolic function [62]. These abnormalities are nonspecific, and normal EchoCG findings cannot exclude the presence of sarcoidosis in a patient. However, transthoracic echocardiography can serve as an excellent screening method for patients with sarcoid granulomas in other organs.

The next method in the physician’s arsenal is magnetic resonance imaging with gadolinium contrast. The main advantage of cardiac MRI in the diagnosis of cardiac sarcoidosis is the detection of foci of late gadolinium enhancement in the myocardium [63]. Findings may include regional wall motion abnormalities with heterogeneous distribution, as well as regional increases in signal intensity at T2 and delayed gadolinium enhancement. The interventricular septum is most commonly involved. However, any segment of the left or right ventricular myocardium may be involved, including the subepicardial, transmural, or myocardial sections [64].

Although cardiac MRI is the gold standard for the diagnosis of cardiac sarcoidosis, it cannot distinguish active from inactive disease [63]. It is also worth noting that magnetic resonance imaging is limited in patients with pacemakers or implantable cardioverter-defibrillators, and the use of gadolinium is contraindicated in patients with advanced renal disease.

18F-FDG-PET in the diagnosis of cardiac sarcoidosis has a few advantages other than a more accurate separation of active and inactive processes, which is important in monitoring the efficacy of therapy [64]. However, whole-body imaging offers the possibility of evaluating extracardiac sarcoidosis, including extrapulmonary sarcoidosis. Macrophages and CD4 T-lymphocytes express glucose transporters on the cell membrane that allow substrates such as FDG to enter cells, which contributes to granulomatous inflammation in sarcoidosis. At present, such findings are mostly incidental [65], as PET is an expensive study.

Both the innate and adaptive immune systems play a crucial role in the pathogenesis of sarcoidosis. Within the innate immune system, NOD-like receptors and Toll-like receptors are of paramount importance, as are cellular factors such as dendritic cells and macrophages. The adaptive immune system, including T-helper 1 (Th1), Th17, regulatory T (Treg), and B-cells, also contributes significantly to the disease process. Sarcoidosis is a polygenic, multifactorial disorder in which various genes alter immune responses to specific antigenic stimuli [66]. In sarcoidosis, an immunological paradox is observed: signs of local inflammation involving type 1 T-helper cells coexist with peripheral energy induced by T-regulatory cells. A hallmark of active sarcoidosis is the predominant expression of interferon-gamma in the affected organs, accompanied by the involvement of active cytokines, such as IL-2, IL-12, and tumor necrosis factor-alpha. The clonal amplification of CD4+ T-cells, typical of sarcoidosis, indicates that a pathogenic antigen contributes to the development of the disease. The development of CD4+ T-cell alveolitis serves as a biomarker that reflects fluctuations in disease activity. Notably, the immune response persists even after the potential antigen/trigger disappears [67].

Reduction of sarcoidosis activity is usually combined with a reduction in alveolitis severity. In typical cases, compact non-caseating epithelioid cell granulomas are formed, which are sterile and located predominantly along the lymphatic outflow pathways in the lungs [13]. The results of clinical studies on the etiology and pathogenesis of sarcoidosis have recently been confirmed in animal models of sarcoidosis [68]. The pathogenesis of the immune response in sarcoidosis involves human heat shock proteins, which can cause sarcoid granuloma formation under the influence of both infectious and non-infectious factors in genetically predisposed individuals. Oxidative stress may play a definite role in the chain of these events [69]. The role of oxidative stress has also been shown in cardiosarcoidosis [70]. In addition, activated macrophages and granuloma cells can produce 1,25-(OH)2-D3 (calcium triol), which leads to hypercalcemia (2–10% of patients) or hypercalciuria (6–30% of patients) and, as a consequence, to urolithiasis and renal failure [71].

On the background of the inability to eliminate the pathogen or chronic autoimmune inflammation in some patients, there is hyperproduction of collagen in the area of granulomas, which further leads to fibrosis and replacement of lung tissue with connective tissue. Fibrotic changes begin at the periphery of granulomas and extend centrally [72,73].

Tregs with mutations in BTNL2 and ANXA11, whose gene products have anti-inflammatory and immune regulatory properties, were found in the biopsy materials of patients with advanced disease. It is possible that Tregs downregulate inflammation and contribute to chronic sarcoidosis [74,75]. Furthermore, we found that Treg cells had a ‘proinflammatory’ phenotype, since CXCR3-expressing Treg cell subsets, including CCR6-CXCR3+ Th1-like and CCR6 + CXCR3+ Th17.1-like Treg cells, were elevated in peripheral blood samples from patients with sarcoidosis compared to healthy controls [76]. Similarly, d’Alessandro et al. found that Treg cells from patients with sarcoidosis expressed high levels of CD103, which plays a central role in T-cell homing to inflamed mucosal tissues [77].

The role of Th2 cells in the progression of sarcoidosis is a subject of active investigation. It has been postulated that a shift from a Th1 to a Th2 cytokine signature may occur in chronic sarcoidosis, possibly as a response to persistent inflammation [76]. Elevated mRNA expression levels of IL-13, a key Th2 cytokine, have been detected in the peripheral blood of sarcoidosis patients [77]. Experiments on laboratory animals [78] and analysis of tissue samples obtained from sarcoidosis patients [79] have demonstrated that hyperproduction of Th2 cytokines is accompanied by the activation and differentiation of tissue macrophages toward an M2 phenotype, which contributes to the development and maintenance of chronic inflammatory foci in tissues, granuloma formation, and fibrosis [80]. Moreover, crosstalk between B-cells and follicular T-helper cells (Tfh) has been observed in chronic sarcoidosis, suggesting that an imbalance in different Tfh cell subsets (particularly increased levels of Tfh2- and Tfh17-like cells, the most effective cell types in upregulating B-cell activation, clonal expansion, and effector plasma cell formation) may play a role in the humoral response and granuloma formation in sarcoidosis [76].

For many years, the role of autoantibodies in the pathogenesis and diagnosis of sarcoidosis has remained unclear. Recent studies have demonstrated the formation of antibodies to vimentin in patients with sarcoidosis [81,82,83]. Khassawneh et al., in their cohort study, showed the formation of autoantibodies to various antigens in 106 patients with pulmonary sarcoidosis and in 120 patients with extrapulmonary sarcoidosis. The authors demonstrated the formation of IgM to vimentin in the peripheral blood in groups with pulmonary and extrapulmonary sarcoidosis, and IgG to vimentin in patients with pulmonary sarcoidosis [81]. Other researchers have shown the formation of IgG and IgA antibodies against vimentin in the bronchoalveolar lavage fluid (BALF) of patients with sarcoidosis. Furthermore, these autoantibodies were higher in BALF compared to the blood serum samples of these patients, which suggests local production of these antibodies in the foci of inflammation in the lungs. Bagavant et al. also identified an increased titer of IgG to vimentin in patients with pulmonary sarcoidosis compared to that in healthy donors [82,83]. However, there are also opposing opinions. For example, Starshinova et al. refuted the significant influence of autoantibodies to vimentin on the pathogenesis of the disease as a whole, despite their presence in patients with sarcoidosis [84]. It was noted, that specific autoantibodies to protein epitopes of various tissues are formed in patients with sarcoidosis. Furthermore, these autoantibodies depend on the involvement of various organs in patients. Thus, there is an increased level of antiretinal antibodies in patients with sarcoidosis and uveitis [85]. Studies of cardiac sarcoidosis have demonstrated the formation of autoantibodies in cardiomyocytes and intercalated discs located between cardiomyocytes in the cardiac tissue [86]. Hanoudi et al. identified autoantibodies as components of the cell cytoskeleton and proteins involved in intracellular lysosomal transport, regardless of the organ affected [87]. In addition, patients with sarcoidosis often have high titers of antinuclear antibodies (ANA) [88] and antibodies against double-stranded DNA (dsDNA) [89]. Shi et al. in their study revealed the formation of anti-citrullinated protein antibodies (ACPA), rheumatoid factor (RF), anti-Sm antibodies, anti-Ro52, anti-Ro60, anti-SSB, anti-P0, anti-CCP, anti-β2-GP, and anti-mitochondrial antibody-M2 in sera from 152 patients with sarcoidosis [90]. In recent years, there has been an active study on the autoimmune serological markers of sarcoidosis. Hanoudi et al. conducted an analysis of tissues obtained from patients with sarcoidosis, where they revealed the formation of autoantibodies to a large number of peptides involved in cellular transport and cytoskeletal components [91]. Recently, based on autoantibodies to the Cofilinμ and Chain A peptides studied by the authors, an epitope-specific immunoassay was developed for the diagnosis of sarcoidosis [92]. However, this immunological panel showed moderate sensitivity and specificity. Thus, despite the progress made in studying the role of autoantibodies in the pathogenesis and diagnosis of various forms of sarcoidosis, this issue still remains open and requires further study.

In pulmonary sarcoidosis, manifestations of the musculoskeletal system may be observed. These manifestations require a differential diagnosis of rheumatological diseases. Thus, in patients with Löfgren’s syndrome, symmetrical bilateral arthritis of the ankle joints is often observed [93]. The occurrence of periarthritis of the ankle joints, which is swelling of the soft tissues adjacent to the joint, has also been described [94,95]. Tenosynovitis and dactylitis of various localizations often appear [96]. Based on the nature of the disease course, arthritis in sarcoidosis can be divided into acute and chronic. Acute arthritis is mainly observed in Löfgren’s syndrome and often resolves spontaneously without sequelae. The most common localizations of acute arthritis and arthralgia in sarcoidosis are ankle joints, knee joints, wrist, and metacarpophalangeal joints [97]. Despite the listed joints, in most cases, oligoarthritis of the ankle joints is bilaterally symmetrical in nature [98] and accompanied by swelling of the joints [99]. It is worth noting that for arthritis in sarcoidosis, tenosynovitis and periarthritis are more typical than true synovitis [100]. The resulting arthritis is often non-erosive in nature, which helps in the differential diagnosis of erosive arthropathy in rheumatological pathologies. It is also worth noting that in sarcoid arthritis, swelling of the periarticular soft tissues predominates without changes in the bones and cartilage [97]. Chronic arthritis is less typical of sarcoidosis and unlike acute arthritis, can progress and cause joint deformations. Usually, more than one joint is involved; oligo- and polyarthritis are common [101], mainly in the joints of the upper extremities [102]. Dactylitis affects both soft tissues and bones and can often manifest as loss of sensitivity and limited movement in the fingers and toes [97]. It is characterized by symmetrical damage to the second and third phalanx, while the metacarpophalangeal joints often remain intact [103]. Dactylitis is accompanied by swelling and erythema, which require a differential diagnosis of psoriatic arthritis [104]. In severe cases of chronic arthritis, patients with sarcoidosis may experience severe joint deformities (Jaccoud arthropathy) [105]. Bone destruction is also uncommon in arthritis in sarcoidosis and erosive changes in the joints. With this type of arthritis, radiographs may reveal periarticular osteoporosis, cystic bone changes with a characteristic lattice appearance [106], erosive lesions of cartilage and bone, and soft tissue edema [97].

Despite the above characteristics of sarcoid arthritis, a recent study of 56 patients with pulmonary sarcoidosis found that 91.1% of patients had arthralgia, and arthritis was observed in only 89.29% of cases. More than half of the patients with pulmonary sarcoidosis had monoarthritis, followed by oligoarthritis, and in fairly rare cases, polyarthritis [107]. These results may be explained by the heterogeneity of the small cohort of patients with sarcoidosis.

Bone involvement can also occur in sarcoidosis. A feature of this pathological process is the formation of bone cysts [108]. On the radiograph of bone sarcoidosis, three types of bone lesions can be distinguished: type 1 is characterized by the formation of large cysts, type 2 is characterized by the formation of multiple small cysts, and type 3 is characterized by the formation of tunnels in the cortex of the bones of the fingers [97]. Cystic lesions of the bones of the hands and feet are known as Perthes-Jungling disease. In bone sarcoidosis, acroosteolysis is also often observed. In addition to cystic lesions, sclerotic lesions are also found in bone sarcoidosis. On X-rays, they exhibit a characteristic lace pattern. Furthermore, in patients with sarcoidosis, it also damages the spinal column, which manifests as lytic, sclerotic, or mixed lesions of the vertebrae [109]. The pathological process is often localized in the lower thoracic and upper lumbar vertebrae [110]. Vertebral sarcoidosis requires a differential diagnosis from a group of spondyloarthritis (for example, axial spondyloarthritis), as well as from bone metastatic processes.

In sarcoidosis, lesions of striated muscles are observed, which manifest as acute myositis, nodular myopathy, or chronic myopathy [110]. Acute myositis clinically manifests as febrile syndrome, polymyalgia, and weakness of the proximal muscles of the upper and lower extremities, which require differential diagnosis with polymyositis. Nodular myopathy is much less common and is characterized by palpable nodules, symmetrical limb involvement, and the absence of laboratory markers of rhabdomyolysis [106,111]. Chronic myopathy is characterized by weakness of the proximal muscles; however, unlike acute myositis, muscle atrophy, the formation of contractures, and fibrosis are also noted. Clinically, it can manifest as muscle pain and muscle weakness, and pain precedes weakness [112].

Thus, the features of damage to the musculoskeletal system in sarcoidosis require differential diagnosis with a wide range of rheumatological pathologies. In some forms of sarcoidosis, there are virtually no pathognomonic signs of damage to the musculoskeletal system; therefore, the key link in this diagnosis is often a biopsy.

Many genes are associated with the development of sarcoidosis. Most of these are associated with impaired immune tolerance. In addition, some of these genes have organ specificity. Thus, alleles associated with the expression of MHC class II required for the presentation of various antigens to T-lymphocytes—HLA-DPA1/DPB1, HLA-DRB1 [113,114], HLA-DRB1*0301, HLA-DRB1*1101, HLA-DRB1*1201 [95], HLA-DQB1, and BTNL2 [115]–represent predispositions to the development of sarcoidosis in various localizations in patients. While the HLA-DRB1*0401 and MAGI1 [116,117] genes represent susceptibility to ocular sarcoidosis. Davoudi et al. in their study demonstrated a predisposition to sarcoid uveitis in patients with genetic variants RAB23, and ANXA11 [113].

At the same time, the HLA-DRB1*04/*15 genes are associated with the risk of developing extrapulmonary manifestations of sarcoidosis, such as lesions of the skin, superficial lymph nodes, eyes, nervous system, kidneys, parotid and salivary glands, heart, liver, spleen, and bone marrow [117]. Other researchers have shown that the HLA-DRB1*0302 gene is associated with the risk of cutaneous sarcoidosis [118]. In a Japanese cohort, a relationship between the HLA-DQB1*0601 allele and the development of cardiac sarcoidosis was identified [119]. These results demonstrate a relationship between the alleles of genes and sarcoidosis phenotypes. However, recent large-scale studies have demonstrated a relationship not only between the sarcoidosis phenotype and genetic variants, but also between the sarcoidosis phenotype and environment [120]. The presented results also represent the influence of external factors on the formation of the sarcoidosis phenotype in patients.

Some allele combinations of the HLA gene can act not only as risk factors for the development of sarcoidosis but also as prognostic factors. Thus, the HLA-DRB1*0301 gene is associated with Löfgren’s syndrome and the resolution of the disease [121]. In another study, the association of genes on chromosome 1p36.21 (AADACL3 and C1orf158) was also associated with disease resolution [122]. Recently, a lot of cohort studies have been conducted to identify the relationship between alleles of various genes and the development of sarcoidosis in relatives of patients with sarcoidosis. Kishore et al., in a genome-wide study of genes in patients with familial pulmonary sarcoidosis, demonstrated 40 functional mutations that are associated with the regulation of immune responses, as well as the regulation of the metabolism of calcium ions and retinoic acid [123]. Among the regulators of calcium ion metabolism, the most important gene that is a risk factor for the development of sarcoidosis is ANXA11. It encodes calcium-dependent membrane-binding proteins, promotes the disruption of apoptosis in sarcoid granuloma cells, and induces autophagy, which supports chronic immune inflammation [124]. Hereditary transmission of pulmonary sarcoidosis was noted by Rossides et al., who conducted a «case-control-family» study of familial sarcoidosis in Swedish families [125]. After analyzing 23,880 patients with sarcoidosis, it was found that having at least one first-degree relative with sarcoidosis was associated with a 3.7-fold increase in the risk of sarcoidosis. Moreover, the risk increased 4.1 times if a relative had Löfgren’s syndrome. The relative risk increased 4.7 times in individuals with two or more relatives with sarcoidosis [124]. Calender et al. identified 37 genes that may be associated with the development of sarcoidosis in children. Among these 37 genes, 9 genes affected autophagy and intracellular transport, 6—on the regulation of G proteins, 4—on T-cell activation, 4—on the cell cycle and regulation of the immune synapse, and 2 genes affected innate immunity [126]. Furthermore, some of these genes have been associated with susceptibility to the development of autoimmune diseases—JAK2, BACH2, and NCF1 [127].

The mTOR signaling pathway and autophagy make major contributions to the pathogenesis of familial sarcoidosis. Calender et al., in their study of familial sarcoidosis in five French families with at least two relatives with sarcoidosis, showed 227 susceptibility variants in 195 genes [128]. Among the identified transcripts, autophagy is the main pathway affected by the above genetic variants. Moreover, mTORC1 and the mTOR complex (MTOR, RICTOR, and MLST8) are the main protein products encoded by the identified transcript variants [129]. In confirmation of this, recent studies have shown activation of the mTORC1 signaling cascade in lesions of skin in a patient with cutaneous sarcoidosis. During treatment with tofacitinib, there was a decrease in mTORC1 mRNA levels along with clinical improvement [130]. In addition, when analyzing biopsy samples of mediastinal lymph nodes in patients with pulmonary sarcoidosis, mTORC1 signaling was found to be activated in granulomas. In addition, Ki-67 expression was increased in these granulomas. Thus, mTORC1-dependent proliferation of macrophages is observed in the granulomas of patients with sarcoidosis, which contributes to the progression of the disease. In a mouse model, it was shown that mTORC1 activation in macrophages caused excessive proliferation and granuloma formation in vivo. A similar effect was demonstrated in vitro, where mTORC1-activated macrophages formed granulomatous structures and showed excessive proliferation mediated by cyclin-dependent kinase 4 (CDK4). In macrophages, mTORC1 promotes the inhibition of apoptosis. Linke et al. also showed that inhibition of mTORC1 can lead to the resolution of sarcoid granulomas in a mouse model [131], which may represent a therapeutic potential for patients with sarcoidosis.

Several factors should be evaluated to initiate treatment: minimizing the risk of disability, mortality, and reduced quality of life, and at the same time, the risk of comorbidities and reduced quality of life due to glucocorticoid and other therapies [132,133,134,135,136,137].

Treatment usually depends on structural changes in the lungs and impaired lung function. While chest radiography and high-resolution computed tomography provide statistical images, hybrid positron emission tomography provides both structural and functional assessments of the lungs. Lung lesions alone are not an indication for treatment, but extensive pulmonary fibrosis increases the long-term risk of respiratory failure [23,138]. Thus, the main indications for initiating systemic treatment are the presence of symptomatic disease affecting the patient’s quality of life and/or the likelihood of disease progression, leading to a significant decline in lung function.

However, no single biomarker of pulmonary sarcoidosis is individually adequate for diagnosis, assessment of disease activity or response to treatment, or prognostic assessment [139].

Unfortunately, to date, no biomarkers or combinations of biomarkers can reliably predict the development of fibrosis. Thus, decisions can be made regarding early aggressive treatment or withdrawal of therapy with close follow-up [21].

## 10. Conclusions

Chronic sarcoidosis is a disease characterized by chronic inflammation and the formation of specific granulomas that can affect various organs and body systems. Despite the large number of studies conducted in this area, many aspects still require further study.

Currently, there are no unambiguously reliable methods for diagnosing changes in sarcoidosis. Radiological diagnostic methods, such as computed tomography, MRI, or positron emission tomography, can determine the extent of inflammatory changes that may characterize sarcoidosis of the heart, lungs, and nervous system.

The features of the course of chronic sarcoidosis may vary depending on the affected organ and the individual characteristics of the patient. There are the most common combinations of organ involvement, but this does not allow for predicting further development of the disease. It is only a reason to be more cautious in the complex examination of a patient with newly diagnosed sarcoidosis, as well as in a patient with long-standing involvement of one organ.

The diagnosis of chronic sarcoidosis is a complex process that requires a multidisciplinary approach that excludes other possible causes of pathological changes.

Currently, no specific biomarker can reliably predict the development and spread of this pathological process. For the most accurate diagnosis and determination of prognosis, the existence of a single serological or imaging marker with sufficient sensitivity and specificity is required. However, the exact etiology and pathophysiology of this process are poorly understood, making it difficult to develop such a marker.

In addition, the diagnosis of sarcoidosis is often difficult due to its diverse course and similarity to other inflammatory and infectious diseases. Overall, this work highlights the importance of further research in the field of chronic sarcoidosis to improve diagnostic methods, understand its course, and develop effective strategies for the follow-up of patients with this disease.

## Figures and Tables

**Table 1:** Results of immunologic study with determination of the level of T- and B-cells.

Populations and Subpopulations of Cells	Relative Numbers of Positive Cells (%)	Absolute Number of Cells Per Liter (×10^9^)		
	Result	Normal Value	Result	Normal Value
Total B-cells (CD3^−^CD19^+^)	29.87	7–17	0.345	0.111–0.376
NK (LGL) (CD3^−^CD16^+^CD56^+^)	8,48	8–17	0.098	0.123–0.369
Total T-cells (CD3^+^CD19^−^)	56.73	61–85	0.655	0.946–2.079
T helpers (CD3^+^CD4^+^)	20.09	35–55	0.336	0.576–1.336
T cytotoxic cells (CD3^+^CD8^+^)	19.39	19–35	0.212	0.372–0.974
NKT cells (CD16^+^CD56^+^CD3^+^)	10.72	0.5–6	0.124	0.007–0.165
Regulatory T-cells (CD4^+^CD25brightCD127neg)	2.1	0.6–3.2	0.025	0.009–0.078
Estimated Indicator				
Indicator	Result		Norma	
Ratio index (Th/Tct)	1.04		1.5–2.6	
Control amount (T-cells + B-cells + NKT)	95.1		100 ± 5	

## References

[1] Vizel A.A., Vizel I.Y., Amirov N.B. (2017). Epidemiology of sarcoidosis in the Russian Federation. Bull. Mod. Clin. Med..

[2] Hena K.M. (2020). Sarcoidosis Epidemiology: Race Matters. Front. Immunol..

[3] Inaoka P.T., Shono M., Kamada M., Espinoza J.L. (2019). Host-microbe interactions in the pathogenesis and clinical course of sarcoidosis. J. Biomed. Sci..

[4] Hena K.M., Yip J., Jaber N., Goldfarb D., Fullam K., Cleven K., Moir W., Zeig-Owens R., Webber M.P., Spevack D.M. (2018). Clinical Course of Sarcoidosis in World Trade Center-Exposed Firefighters. Chest.

[5] Bennett D., Bargagli E., Refini R.M., Rottoli P. (2019). New concepts in the pathogenesis of sarcoidosis. Expert. Rev. Respir. Med..

[6] Malkova A., Starshinova A., Zinchenko Y., Gavrilova N., Kudryavtsev I., Lapin S., Mazing A., Surkova E., Pavlova M., Belaeva E. (2021). New laboratory criteria of the autoimmune inflammation in pulmonary sarcoidosis and tuberculosis. Clin. Immunol..

[7] Bagavant H., Cizio K., Araszkiewicz A.M., Papinska J.A., Garman L., Li C., Pezant N., Drake W.P., Montgomery C.G., Deshmukh U.S. (2022). Systemic immune response to vimentin and granuloma formation in a model of pulmonary sarcoidosis. J. Transl. Autoimmun..

[8] Cozier Y.C., Govender P., Berman J.S. (2018). Obesity and sarcoidosis: Consequence or contributor?. Curr. Opin. Pulm. Med..

[9] Crouser E.D., Maier L.A., Wilson K.C., Bonham C.A., Morgenthau A.S., Patterson K.C., Abston E., Bernstein R.C., Blankstein R., Chen E.S. (2020). Diagnosis and Detection of Sarcoidosis. An Official American Thoracic Society Clinical Practice Guideline. Am. J. Respir. Crit. Care Med..

[10] Schupp J.C., Freitag-Wolf S., Bargagli E., Mihailović-Vučinić V., Rottoli P., Grubanovic A., Müller A., Jochens A., Tittmann L., Schnerch J. (2018). Phenotypes of organ involvement in sarcoidosis. Eur. Respir. J..

[11] Rosen Y. (2015). Four decades of necrotizing sarcoid granulomatosis: What do we know now?. Arch. Pathol. Lab. Med..

[12] Baughman R.P., Teirstein A.S., Judson M.A., Rossman M.D., Yeager H., Bresnitz E.A., DePalo L., Hunninghake G., Iannuzzi M.C., Johns C.J. (2001). Case Control Etiologic Study of Sarcoidosis (ACCESS) research group. Clinical characteristics of patients in a case control study of sarcoidosis. Am. J. Respir. Crit. Care Med..

[13] Polverino F., Balestro E., Spagnolo P. (2020). Clinical Presentations, Pathogenesis, and Therapy of Sarcoidosis: State of the Art. J. Clin. Med..

[14] Mañá J., Rubio-Rivas M., Villalba N., Marcoval J., Iriarte A., Molina-Molina M., Llatjos R., García O., Martínez-Yélamos S., Vicens-Zygmunt V. (2017). Multidisciplinary approach and long-term follow-up in a series of 640 consecutive patients with sarcoidosis: Cohort study of a 40-year clinical experience at a tertiary referral center in Barcelona, Spain. Medicine.

[15] Ungprasert P., Matteson E.L. (2017). Neurosarcoidosis. Rheum. Dis. Clin. N. Am..

[16] Bargagli E., Prasse A. (2018). Sarcoidosis: A review for the internist. Intern. Emerg. Med..

[17] Jameson A., Revels J., Wang L.L., Wang D.T., Wang S.S. (2022). Sarcoidosis, the master mimicker. Curr. Probl. Diagn. Radiol..

[18] Castro M.D.C., Pereira C.A.C. (2020). Nonlife-Threatening Sarcoidosis. Semin. Respir. Crit. Care Med..

[19] Kharoubi S. (2020). Heerfordt’s syndrome: About a case and literature review. Pan Afr. Med. J..

[20] Mikulic S., Patel P., Sheffield S., Kandah F., Velarde G. (2020). Heerfordt-Waldenström Syndrome Manifesting as Cardiac Sarcoidosis. Cureus.

[21] Trivieri M.G., Spagnolo P., Birnie D., Liu P., Drake W., Kovacic J.C., Baughman R., Fayad Z.A., Judson M.A. (2020). Challenges in Cardiac and Pulmonary Sarcoidosis: JACC State-of-the-Art Review. J. Am. Coll. Cardiol..

[22] Obi O.N., Saketkoo L.A., Russell A.-M., Baughman R.P. (2022). Sarcoidosis: Updates on therapeutic drug trials and novel treatment approaches. Front. Med..

[23] Baughman R.P., Valeyre D., Korsten P., Mathioudakis A.G., Wuyts W.A., Wells A., Rottoli P., Nunes H., Lower E.E., Judson M.A. (2021). ERS clinical practice guidelines on treatment of sarcoidosis. Eur. Respir. J..

[24] Sawahata M., Yamaguchi T. (2022). Imaging Findings of Fibrosis in Pulmonary Sarcoidosis. Sarcoidosis Vasculitis Diffuse Lung Dis. Off. J. WASOG.

[25] Tana C., Donatiello I., Caputo A., Tana M., Naccarelli T., Mantini C., Ricci F., Ticinesi A., Meschi T., Cipollone F. (2021). Clinical Features, Histopathology and Differential Diagnosis of Sarcoidosis. Cells.

[26] Levy A., Hamzeh N., Maier L.A. (2018). Is it time to scrap Scadding and adopt computed tomography for initial evaluation of sarcoidosis?. F1000Research.

[27] Nishino M., Lee K.S., Itoh H., Hatabu H. (2010). The spectrum of pulmonary sarcoidosis: Variations of high-resolution CT findings and clues for specific diagnosis. Eur. J. Radiol..

[28] Polychronopoulos V.S., Prakash U.B.S. (2009). Airway involvement in sarcoidosis. Chest.

[29] Jeny F., Bernaudin J.-F., Aubart F.C., Brillet P.-Y., Bouvry D., Nunes H., Valeyre D. (2020). Diagnosis issues in sarcoidosis. Respir. Med. Res..

[30] Valeyre D., Brauner M., Bernaudin J.-F., Carbonnelle E., Duchemann B., Rotenberg C., Berger I., Martin A., Nunes H., Naccache J.-M. (2023). Differential diagnosis of pulmonary sarcoidosis: A review. Front. Med..

[31] Mankad P., Mitchell B., Birnie D., Kron J. (2019). Cardiac Sarcoidosis. Curr. Cardiol. Rep..

[32] Birnie D.H. (2020). Cardiac Sarcoidosis. Semin. Respir. Crit. Care Med..

[33] Terasaki F., Kusano K., Nakajima T., Yazaki Y., Morimoto S.I., Culver D.A., Isobe M. (2022). The characteristics of Japanese guidelines on diagnosis and treatment of cardiac sarcoidosis compared with the previous guidelines. Sarcoidosis Vasculitis Diffuse Lung Dis. Off. J. WASOG.

[34] Liu J., Ma P., Lai L., Villanueva A., Koenig A., Bean G.R., Bowles D.E., Glass C., Watson M., Lavine K.J. (2022). Transcriptional and Immune Landscape of Cardiac Sarcoidosis. Circ. Res..

[35] Birnie D.H., Sauer W.H., Bogun F., Cooper J.M., Culver D.A., Duvernoy C.S., Judson M.A., Kron J., Mehta D., Nielsen J.C. (2014). HRS expert consensus statement on the diagnosis and management of arrhythmias associated with cardiac sarcoidosis. Heart Rhythm..

[36] Hulten E., Agarwal V., Cahill M., Cole G., Vita T., Parrish S., Bittencourt M.S., Murthy V.L., Kwong R., Di Carli M.F. (2016). Presence of Late Gadolinium Enhancement by Cardiac Magnetic Resonance Among Patients With Suspected Cardiac Sarcoidosis Is Associated With Adverse Cardiovascular Prognosis: A Systematic Review and Meta-Analysis. Circulation. Cardiovasc. Imaging.

[37] Sperry B.W., Tamarappoo B.K., Oldan J.D., Javed O., Culver D.A., Brunken R., Cerqueira M.D., Hachamovitch R. (2018). Prognostic Impact of Extent, Severity, and Heterogeneity of Abnormalities on 18F-FDG PET Scans for Suspected Cardiac Sarcoidosis. JACC. Cardiovasc. Imaging.

[38] Silverstein A., Siltzbach L.E. (1969). Muscle involvement in sarcoidosis. Asymptomatic, myositis, and myopathy. Arch Neurol..

[39] Nordenswan H.-K., Pöyhönen P., Lehtonen J., Ekström K., Uusitalo V., Niemelä M., Vihinen T., Kaikkonen K., Haataja P., Kerola T. (2022). Incidence of Sudden Cardiac Death and Life-Threatening Arrhythmias in Clinically Manifest Cardiac Sarcoidosis With and Without Current Indications for an Implantable Cardioverter Defibrillator. Circulation.

[40] Caplan A., Rosenbach M., Imadojemu S. (2020). Cutaneous Sarcoidosis. Semin. Respir. Crit. Care Med..

[41] Boch K., Langan E.A., Zillikens D., Ludwig R.J., Kridin K. (2022). Evaluation of clinical and laboratory characteristics of patients with cutaneous sarcoidosis: A single-center retrospective cohort study. Front. Med..

[42] Alghamdi A., Mazraani N., Thabet S.A., Alghamdi B.S., Hanawi M., Almaghraby H., Huwait H.F. (2021). Cutaneous Sarcoidosis of a 53-Year-Old Female: A Case Report. Cureus.

[43] Arora P., Verma G., Chauhan M., Ahuja A. (2022). Atypical Protean Manifestations of Cutaneous Sarcoidosis. Indian. J. Dermatol..

[44] Tsai H.L., Chang J.W., Lu J.H., Liu C.S. (2022). Epidemiology and risk factors associated with avascular necrosis in patients with autoimmune diseases: A nationwide study. Korean J. Intern. Med..

[45] Barreras P., Stern B.J. (2022). Clinical features and diagnosis of neurosarcoidosis-review article. J. Neuroimmunol..

[46] Bradshaw M.J., Pawate S., Koth L.L., Cho T.A., Gelfand J.M. (2021). Neurosarcoidosis: Pathophysiology, Diagnosis, and Treatment. Neurol. (R) Neuroimmunol. Neuroinflammation.

[47] Arun T., Palace J. (2021). Effects of immunotherapies and clinical outcomes in neurosarcoidosis: A retrospective cohort study. J. Neurol..

[48] Shen J., Lackey E., Shah S. (2023). Neurosarcoidosis: Diagnostic Challenges and Mimics A Review. Curr. Allergy Asthma Rep..

[49] Tavee J., Voortman M. (2022). Granulomatous and nongranulomatous neurological sarcoidosis. Eur. Respir. Soc. Monogr..

[50] Mekinian A., Maisonobe L., Boukari L., Melenotte C., Terrier B., Ayrignac X., Schleinitz N., Sène D., Hamidou M., Konaté A. (2018). Characteristics, outcome and treatments with cranial pachymeningitis: A multicenter French retrospective study of 60 patients. Medicine.

[51] Stern B.J., Royal W., Gelfand J.M., Clifford D.B., Tavee J., Pawate S., Berger J.R., Aksamit A.J., Krumholz A., Pardo C.A. (2018). Definition and Consensus Diagnostic Criteria for Neurosarcoidosis: From the Neurosarcoidosis Consortium Consensus Group. JAMA Neurol..

[52] Voortman M., Drent M., Baughman R.P. (2019). Management of neurosarcoidosis: A clinical challenge. Curr. Opin. Neurol..

[53] Giorgiutti S., Jacquot R., El Jammal T., Bert A., Jamilloux Y., Kodjikian L., Sève P. (2023). Sarcoidosis-Related Uveitis: A Review. J. Clin. Med..

[54] Bazewicz M., Heissigerova J., Pavesio C., Willermain F., Skrzypecki J. (2023). Ocular sarcoidosis in adults and children: Update on clinical manifestation and diagnosis. J. Ophthalmic Inflamm. Infect..

[55] Standardization of Uveitis Nomenclature (SUN) Working Group (2021). Classification Criteria for Sarcoidosis-Associated Uveitis. Am. J. Ophthalmol..

[56] Sève P., Pacheco Y., Durupt F., Jamilloux Y., Gerfaud-Valentin M., Isaac S., Boussel L., Calender A., Androdias G., Valeyre D. (2021). Sarcoidosis: A Clinical Overview from Symptoms to Diagnosis. Cells.

[57] Takase H. (2022). Characteristics and management of ocular sarcoidosis. Immunol. Med..

[58] Akao K., Minezawa T., Yamamoto N., Okamura T., Inoue T., Yamatsuta K., Uozu S., Goto Y., Hayashi M., Isogai S. (2018). Flow cytometric analysis of lymphocyte profiles in mediastinal lymphadenopathy of sarcoidosis. PLoS ONE.

[59] Kraaijvanger R., Bonás M.J., Vorselaars A.D.M., Veltkamp M. (2020). Biomarkers in the Diagnosis and Prognosis of Sarcoidosis: Current Use and Future Prospects. Front. Immunol..

[60] Ramos-Casals M., Retamozo S., Sisó-Almirall A., Pérez-Alvarez R., Pallarés L., Brito-Zerón P. (2019). Clinically-useful serum biomarkers for diagnosis and prognosis of sarcoidosis. Expert Rev. Clin. Immunol..

[61] Zhou Y., Chen X., Zhao M., Lower E.E., Baughman R.P. (2023). SACE and IL-2R as serum biomarkers for evaluation of multi-organ involvement and prognosis of sarcoidosis. Respir. Res..

[62] Ramirez R., Trivieri M., Fayad Z.A., Ahmadi A., Narula J., Argulian E. (2019). Advanced Imaging in Cardiac Sarcoidosis. Journal of nuclear medicine: Official publication. Soc. Nuclear Med..

[63] Al Hayja M.A., Vinjamuri S. (2023). Cardiac sarcoidosis: The role of cardiac MRI and 18F-FDG-PET/CT in the diagnosis and treatment follow-up. Br. J. Cardiol..

[64] Strambu I.R. (2023). Challenges of cardiac sarcoidosis. Front. Med..

[65] Vender R.J., Aldahham H., Gupta R. (2022). The role of PET in the management of sarcoidosis. Curr. Opin. Pulm. Med..

[66] Starshinova A., Malkova A., Zinchenko U., Lapin S., Mazing A., Kudlay D., Yablonskiy P., Shoenfeld Y. (2022). Detection of Anti-Vimentin Antibodies in Patients with Sarcoidosis. Diagnostics.

[67] Baughman R.P., Culver D.A., Judson M.A. (2011). A concise review of pulmonary sarcoidosis. Am. J. Respir. Crit. Care Med..

[68] Patterson K.C., Chen E.S. (2018). The pathogenesis of pulmonary sarcoidosis and implications for treatment. Chest.

[69] Hu Y., Yibrehu B., Zabini D., Kuebler W.M. (2017). Animal models of sarcoidosis. Cell. Tissue Res..

[70] Dubaniewicz A. (2018). “Danger theory”as a common mechanism of sarcoidosis induction by infectious and non- infectious factors-a role of environmental factors and autoimmunity. Pol. Merkur. Lekarski.

[71] Jouni H., Chareonthaitawee P. (2017). Unraveling inflammation and oxidative stress in cardiac sarcoidosis. Circ. Cardiovasc. Imaging.

[72] Ruža I., Lucāne Z. (2018). Serum and urinary calcium level in Latvian patients with sarcoidosis. Reumatologia.

[73] Starshinova A.A., Malkova A.M., Basantsova N.Y., Zinchenko Y.S., Kudryavtsev I.V., Ershov G.A., Soprun L.A., Mayevskaya V.A., Churilov L.P., Yablonskiy P.K. (2020). Sarcoidosis as an Autoimmune Disease. Front. Immunol..

[74] Puttgen K.B. (2014). Diagnosis and management of of infantile hemangiomas. Pediatr. Clin. N. Am..

[75] Berge B.T., KleinJan A., Muskens F., Hammad H., Hoogsteden H.C., Hendriks R.W., Lambrecht B.N., Van den Blink B. (2012). Evidence for local dendritic cell activation in pulmonary sarcoidosis. Respir. Res..

[76] Sakthivel P., Bruder D. (2017). Mechanism of granuloma formation in sarcoidosis. Curr. Opin. Hematol..

[77] Broos C.E., van Nimwegen M., Hoogsteden H.C., Hendriks R.W., Kool M., Van den Blink B. (2013). Granuloma formation in pulmonary sarcoidosis. Front. Immunol..

[78] Oliver S.J., Kikuchi T., Krueger J.G., Kaplan G. (2002). Thalidomide induces granuloma differentiation in sarcoid skin lesions associated with disease improvement. Clin. Immunol..

[79] Kita S., Tsuda T., Sugisaki K., Miyazaki E., Matsumoto T. (1995). Characterization of Distribution of T Lymphocyte Subsets and Activated T Lymphocytes Infiltrating into Sarcoid Lesions. Intern. Med..

[80] Crouser E. (2018). Role of imbalance between Th17 and regulatory T-cells in sarcoidosis. Curr. Opin. Pulm. Med..

[81] Khassawneh B., Zhu C., Barkes B., Vestal B., Shrock S., Gillespie M., Pacheco K., Deane K.D., Maier L.A., Li Q.-Z. (2022). Autoantibody profile in sarcoidosis, analysis from the GRADS sarcoidosis cohort. PLoS ONE.

[82] Cain H., Kraus B. (1983). Immunofluorescence microscopic demonstration of vimentin filaments in asteroid bodies of sarcoidosis. A comparison with electron microscopic findings. Virchows Arch. B Cell. Pathol. Incl. Mol. Pathol..

[83] Kinloch A.J., Kaiser Y., Wolfgeher D., Ai J., Eklund A., Clark M.R., Grunewald J. (2018). In Situ Humoral Immunity to Vimentin in HLA-DRB1*03+ Patients with Pulmonary Sarcoidosis. Front. Immunol..

[84] Weeratunga P., Moller D.R., Ho L.P. (2024). Immune mechanisms of granuloma formation in sarcoidosis and tuberculosis. J. Clin. Investig..

[85] Avendaño-Monje C.L., Cordero-Coma M., Mauriz J.L., Calleja-Antolín S., Fonollosa A., Llordén A.G., García-Sancho J.M., Sánchez-Salazar M.I., de Morales J.G.R. (2022). Anti-retinal Antibodies in Sarcoidosis. Ocul. Immunol. Inflamm..

[86] Caforio A.L.P., Baritussio A., Marcolongo R., Cheng C.-Y., Pontara E., Bison E., Cattini M.G., Gallo N., Plebani M., Iliceto S. (2021). Serum Anti-Heart and Anti-Intercalated Disk Autoantibodies: Novel Autoimmune Markers in Cardiac Sarcoidosis. J. Clin. Med..

[87] Hanoudi S.N., Talwar H., Draghici S., Samavati L. (2022). Autoantibodies against cytoskeletons and lysosomal trafficking discriminate sarcoidosis from healthy controls, tuberculosis and lung cancers. Mol. Biomed..

[88] Ueda-Hayakawa I., Tanimura H., Osawa M., Iwasaka H., Ohe S., Yamazaki F., Mizuno K., Okamoto H. (2013). Elevated serum BAFF levels in patients with sarcoidosis: Association with disease activity. Rheumatology.

[89] Weinberg I., Vasiliev L., Gotsman I. (2000). Anti-dsDNA antibodies in sarcoidosis. Semin. Arthritis Rheum..

[90] Shi T.-Y., Wen X.-H., Shi X.-H., Meng J., Lu Y.-W. (2022). Associations between sarcoidosis, autoimmune diseases, and autoantibodies: A single-center retrospective study in China. Clin. Exp. Med..

[91] Maertzdorf J., Weiner J., Mollenkopf H.J., Bauer T., Prasse A., Müller-Quernheim J., Kaufmann S.H., TBornotTB Network (2012). Common patterns and disease-related signatures in tuberculosis and sarcoidosis. Proc. Natl. Acad. Sci. USA.

[92] Peng C., Talreja J., Steinbauer B., Shinki K., Koth L.L., Samavati L. (2024). Discovery of Two Novel Immunoepitopes and Development of Peptide-based Sarcoidosis Immunoassay. Am. J. Respir. Crit. Care Med..

[93] Thillai M., Atkins C.P., Crawshaw A., Hart S.P., Ho L.-P., Kouranos V., Patterson K.C., Screaton N.J., Whight J., Wells A.U. (2021). BTS Clinical Statement on pulmonary sarcoidosis. Thorax.

[94] Starshinova A., Zinchenko Y., Filatov M., Denisova N., Istomina E., Landa S., Burdakov V., Churilov L., Sapozhnikova N., Pavlova M. (2018). Specific features of immune complexes in patients with sarcoidosis and pulmonary tuberculosis. Immunol. Res..

[95] Grunewald J., Eklund A. (2007). Sex-specific manifestations of Löfgren’s syndrome. Am. J. Respir. Crit. Care Med..

[96] Kellner H., Späthling S., Herzer P. (1992). Ultrasound findings in Löfgren’s syndrome: Is ankle swelling caused by arthritis. tenosynovitis or periarthritis?. J. Rheumatol..

[97] Kobak S. (2015). Sarcoidosis: A rheumatologist’s perspective. Ther. Adv. Musculoskelet. Dis..

[98] Koilubaeva G., Egorova O., Bolotbekova A., Turatbekova A., Syunbai K G. (2023). Ab1700 rheumatologist’s assessment of acute sarcoidosis. Ann. Rheum. Dis..

[99] Visser H., Vos K., Zanelli E., Verduyn W., Schreuder G.M.T., Speyer I., Breedveld F.C., Hazes J.M.W. (2002). Sarcoid arthritis: Clinical characteristics, diagnostic aspects, and risk factors. Ann. Rheum. Dis..

[100] Bechman K., Christidis D., Walsh S., Birring S.S., Galloway J. (2018). A review of the musculoskeletal manifestations of sarcoidosis. Rheumatology.

[101] Smedslund G., Kotar A.M., Uhlig T. (2022). Sarcoidosis with musculoskeletal manifestations: Systematic review of non-pharmacological and pharmacological treatments. Rheumatol. Int..

[102] Yeung T., Grebowicz A., Nevskaya T., Zahid S., Pope J.E. (2024). Joint involvement in sarcoidosis: Systematic review and meta-analysis of prevalence, clinical pattern and outcome. Rheumatology.

[103] Awada H., Abi-Karam G., Fayad F. (2003). Musculoskeletal and other extrapulmonary disorders in sarcoidosis. Best. Pr. Pract. Res. Clin. Rheumatol..

[104] Rothschild B.M., Pingitore C., Eaton M. (1998). Dactylitis: Implications for clinical practice. Semin. Arthritis Rheum..

[105] Sawada K., Segal A.M., Malchesky P.S., Koo A.P., Naganuma S., Nosé Y. (1991). Rapid improvement in a patient with leukocytoclastic vasculitis with secondary mixed cryoglobulinemia treated with cryofiltration. J. Rheumatol..

[106] Brill A.K., Ott S.R., Geiser T. (2013). Effect and safety of mycophenolate mofetil in chronic pulmonary sarcoidosis: A retrospective study. Respiration.

[107] Karabulut Y., Öz N., Gezer H.H., Esen I., Duruöz M.T. (2022). Perspective of sarcoidosis in terms of rheumatology: A single-center rheumatology clinic experience. Rheumatol. Int..

[108] Shorr A.F., Murphy F.T., Kelly W.F., Kaplan K.J., Gilliland W.R., Shapeero L.G. (1998). Osseous sarcoidosis clinical, radiographic, and therapeutic observations. J. Clin. Rheumatol..

[109] Rúa-Figueroa I., Gantes M.A., Erausquin C., Mhaidli H., Montesdeoca A. (2002). Vertebral sarcoidosis: Clinical and imaging findings. Semin. Arthritis Rheum..

[110] Valeyre D., Prasse A., Nunes H., Uzunhan Y., Brillet P.Y., Müller-Quernheim J. (2014). Sarcoidosis. Lancet.

[111] Nemoto I., Shimizu T., Fujita Y., Tateishi Y., Tsuji-Abe Y., Shimizu H. (2007). Tumour-like muscular sarcoidosis. Clin. Exp. Dermatol..

[112] Colebunders R., Mercelis R., Buyssens N., Martin J.J. (1984). Symptomatic sarcoid myopathy with minimal involvement of the peripheral nerves. Acta Clin. Belg..

[113] Cleven K.L., Ye K., Zeig-Owens R., Hena K.M., Montagna C., Shan J., Hosgood H.D., Jaber N., Weiden M.D., Colbeth H.L. (2019). Genetic Variants Associated with FDNY WTC-Related Sarcoidosis. Int. J. Environ. Res. Public Health.

[114] Rossman M.D., Thompson B., Frederick M., Maliarik M., Iannuzzi M.C., Rybicki B.A., Pandey J.P., Newman L.S., Magira E., Beznik-Cizman B. (2003). HLA-DRB1*1101: A significant risk factor for sarcoidosis in blacks and whites. Am. J. Hum. Genet..

[115] Spagnolo P., Maier L.A. (2021). Genetics in sarcoidosis. Curr. Opin. Pulm. Med..

[116] Garman L., Pezant N., Pastori A., Savoy K.A., Li C., Levin A.M., Iannuzzi M.C., Rybicki B.A., Adrianto I., Montgomery C.G. (2021). Genome-Wide Association Study of Ocular Sarcoidosis Confirms HLA Associations and Implicates Barrier Function and Autoimmunity in African Americans. Ocul. Immunol. Inflamm..

[117] Davoudi S., Chang V.S., Navarro-Gomez D., Stanwyck L.K., Sevgi D.D., Papavasileiou E., Ren A., Uchiyama E., Sullivan L., Lobo A.M. (2018). Association of genetic variants in RAB23 and ANXA11 with uveitis in sarcoidosis. Mol. Vis..

[118] Darlington P., Gabrielsen A., Sörensson P., Tallstedt L., Padyukov L., Eklund A., Grunewald J. (2014). HLA-alleles associated with increased risk for extra-pulmonary involvement in sarcoidosis. Tissue Antigens.

[119] Calender A., Weichhart T., Valeyre D., Pacheco Y. (2020). Current Insights in Genetics of Sarcoidosis: Functional and Clinical Impacts. J. Clin. Med..

[120] Naruse T., Matsuzawa Y., Ota M., Katsuyama Y., Matsumori A., Hara M., Nagai S., Morimoto S., Sasayama S., Inoko H. (2000). HLA-DQB1*0601 is primarily associated with the susceptibility to cardiac sarcoidosis. Tissue Antigens.

[121] Freitag-Wolf S., Schupp J.C., Frye B.C., Fischer A., Anwar R., Kieszko R., Mihailović-Vučinić V., Milanowski J., Jovanovic D., Zissel G. (2023). Genetic and geographic influence on phenotypic variation in European sarcoidosis patients. Front. Med..

[122] Gerke A.K., Hunninghake G. (2008). The Immunology of Sarcoidosis. Clin. Chest Med..

[123] Lahtela E., Kankainen M., Sinisalo J., Selroos O., Lokki M.L. (2019). Exome Sequencing Identifies Susceptibility Loci for Sarcoidosis Prognosis. Front. Immunol..

[124] Kishore A., Petersen B.-S., Nutsua M., Müller-Quernheim J., Franke A., Fischer A., Schreiber S., Petrek M. (2018). Whole-exome sequencing identifies rare genetic variations in German families with pulmonary sarcoidosis. Hum. Genet..

[125] Zhou H., Diao M., Zhang M. (2016). The Association between ANXA11 Gene Polymorphisms and Sarcoidosis: A Meta-Analysis and systematic review. Sarcoidosis Vasc. Diffus. Lung Dis..

[126] Rossides M., Grunewald J., Eklund A., Kullberg S., Di Giuseppe D., Askling J., Arkema E.V. (2018). Familial aggregation and heritability of sarcoidosis: A Swedish nested case-control study. Eur. Respir. J..

[127] Calender A., France I.T.F.O.G.S., Farnier P.A.R., Buisson A., Pinson S., Bentaher A., Lebecque S., Corvol H., Taam R.A., Houdouin V. (2018). Whole exome sequencing in three families segregating a pediatric case of sarcoidosis. BMC Med. Genom..

[128] Fritz D., Ferwerda B., Brouwer M.C., van de Beek D. (2021). Whole genome sequencing identifies variants associated with sarcoidosis in a family with a high prevalence of sarcoidosis. Clin. Rheumatol..

[129] Calender A., Lim C.X., Weichhart T., Buisson A., Besnard V., Rollat-Farnier P.A., Bardel C., Roy P., Cottin V., Devouassoux G. (2019). Exome sequencing and pathogenicity-network analysis of five French families implicate mTOR signalling and autophagy in familial sarcoidosis. Eur. Respir. J..

[130] Cinetto F., Scarpa R., Dell’Edera A., Jones M.G. (2020). Immunology of sarcoidosis: Old companions, new relationships. Curr. Opin. Pulm. Med..

[131] Damsky W., Thakral D., Emeagwali N., Galan A., King B. (2018). Tofacitinib Treatment and Molecular Analysis of Cutaneous Sarcoidosis. N. Engl. J. Med..

[132] Linke M., Pham H.T.T., Katholnig K., Schnöller T., Miller A., Demel F., Schütz B., Rosner M., Kovacic B., Sukhbaatar N. (2017). Chronic signaling via the metabolic checkpoint kinase mTORC1 induces macrophage granuloma formation and marks sarcoidosis progression. Nat. Immunol..

[133] Wilson J.L., Mayr H.K., Weichhart T. (2019). Metabolic Programming of Macrophages: Implications in the Pathogenesis of Granulomatous Disease. Front. Immunol..

[134] Shamaei M., Mortaz E., Pourabdollah M., Garssen J., Tabarsi P., Velayati A., Adcock I.M. (2018). Evidence for M2 macrophages in granulomas from pulmonary sarcoidosis: A new aspect of macrophage heterogeneity. Hum. Immunol..

[135] Braun N.A., Celada L.J., Herazo-Maya J.D., Abraham S., Shaginurova G., Sevin C.M., Grutters J., Culver D.A., Dworski R., Sheller J. (2014). Blockade of the programmed death-1 pathway restores sarcoidosis CD4+ T-cell proliferative capacity. Am. J. Respir. Crit. Care Med..

[136] Valentonyte R., Hampe J., Huse K., Rosenstiel P., Albrecht M., Stenzel A., Nagy M., Gaede K.I., Franke A., Haesler R. (2005). Sarcoidosis is associated with a truncating splice site mutation in BTNL2. Nat. Genet..

[137] Hofmann S., Franke A., Fischer A., Jacobs G., Nothnagel M., Gaede K.I., Schürmann M., Müller-Quernheim J., Krawczak M., Rosenstiel P. (2008). Genome-wide association study identifies ANXA11 as a new susceptibility locus for sarcoidosis. Nat. Genet..

[138] Hauber H.P., Gholami D., Meyer A., Pforte A. (2003). Increased interleukin-13 expression in patients with sarcoidosis. Thorax.

[139] Locke L.W., Crouser E.D., White P., Julian M.W., Caceres E.G., Papp A.C., Le V.T., Sadee W., Schlesinger L.S. (2019). IL-13–regulated Macrophage Polarization during Granuloma Formation in an In Vitro Human Sarcoidosis Model. Am. J. Respir. Cell Mol. Biol..

